# Supplementary material for: Helical sensors of membrane saturation: Changes in orientation and curvature preference
Source: Biophys J. 2025 Oct 3;124(23):4087–95. doi: 10.1016/j.bpj.2025.09.042 (PMC12709426; doi:10.1016/j.bpj.2025.09.042)
Supplement: Document S1. Figures S1–S17 and Table S1 [file mmc1.pdf]

Biophysical Journal, Volume *124*

Supplemental information

Helical sensors of membrane saturation: Changes in orientation and curvature preference

Sushmita Pal, Peter Pajtinka, Matti Javanainen, and Robert Vácha

Helical Sensors of Membrane Saturation: Changes in Orientation and Curvature Preference

Sushmita Pal[1,2], Peter Pajtinka[1,2], Matti Javanainen[4,5], and Robert Vácha[1,2,3,*]

[1]CEITEC – Central European Institute of Technology, Masaryk University, Kamenice 753/5, 625 00 Brno, Czech Republic
[2]National Centre for Biomolecular Research, Faculty of Science, Masaryk University, Kamenice 5, 625 00 Brno, Czech Republic
[3]Department of Condensed Matter Physics, Faculty of Science, Masaryk University, Kotlářská 267/2, 611 37 Brno, Czech Republic
[4]Unit of Physics, Tampere University, FI-33720 Tampere, Finland
[5]Institute of Biotechnology, University of Helsinki, FI-00790 Helsinki, Finland
[*]Correspondence: robert.vacha@muni.cz

SUPPLEMENTARY METHODS

Insertion Depth

The system with planar membrane comprised of 184 lipid molecules (92 per leaflet) and two peptide copies, one copy placed on each leaflet in the headgroup region with parallel orientation to the membrane plane. The system contained approximately 3,000 water beads. To mimic physiological conditions, Na and Cl ions were added at a 0.15 M concentration. The energy of the system was minimized by the steepest descent algorithm.

The system preparation proceeded with a 100 ns equilibration run with a timestep of 20 femtoseconds, where the temperature was kept at 310 K using a stochastic velocity rescaling thermostat (1) with a coupling constant of 1 ps. The pressure was maintained at 1 bar using the Berendsen (2) barostat with semi-isotropic coupling and a coupling constant of 5 ps using a compressibility of 3×10^{-4} bar^{-1}. Electrostatic interactions were screened by a dielectric constant of 15, and treated using a reaction field method for speed (3), although long-range contributions were eliminated by setting $\epsilon_{RF} = \infty$ beyond a cutoff radius of 1.1 nm. The cutoff radius for the van der Waals interactions was also set to 1.1 nm. The molecular dynamics simulations were performed at temperature 310 K using the stochastic velocity rescaling algorithm (1) with a time constant of 1 ps. Semi-isotropic Parinello–Rahman (4) barostat was used to maintain the pressure of 1 bar with coupling constant of 12 ps and a compressibility set to 3×10^{-4} bar^{-1}. The rest of the settings were identical to those used in the NPT equilibration.

The reported lipid packing defects (Figure S3) were computed using the PackMem package (5). The values represent the total defect area calculated from Martini coarse-grained simulations, reflecting two-dimensional surface defects where hydrophobic regions are transiently exposed to the solvent (5). While the CG defect areas computed using the Martini force field exhibit a qualitative correlation with defect trends observed in all-atom simulations, the absolute values of the defect areas are not directly comparable. This is due to the intrinsic resolution limitation of CG models, where each bead represents multiple atoms, leading to spatial averaging. Therefore, the defect area computed in Martini simulations should be interpreted as an indicative metric, useful for comparative analysis across systems, but not as a precise quantitative measure of solvent-exposed area.

We have analyzed spatially resolved properties—including bilayer thickness and lipid order parameters—around the peptide insertion site. The thickness was calculated with considering a local cylinder around the peptide of radius 2.5 nm and then the thickness was measured by taking an average z-distance between the phosphate beads between the two leaflets. The order parameter was calculated using the gorder package to see the local effects of peptide on altering the order parameter of the lipids (6).

Free energy calculations

A planar system with 400 lipids of type POPC, DOPC and PAPC were using CHARMM-GUI web server. These membranes were then subjected to energy minimization employing the steepest descent algorithm, followed by an equilibration protocol with varying timesteps and duration, (i) 2 fs and 1 ns, (ii) 5 fs and 1 ns, (iii) 10 fs and 0.1 ns, (iv) 15 fs and 0.45 ns, and (v) 20 fs and 0.1 ns and a longer equilibration of 3 ns with 20 fs timestep. Prior to the addition of peptides, both solvent and ions were removed from the system. One peptide was placed on upper membrane leaflet. Subsequently, the system was resolvated with approximately 12,000 water beads and ions were added to reach the concentration of 0.15 M NaCl, replicating physiological conditions.

The energy minimization of the system was performed again using the steepest descent algorithm, setting the total energy tolerance to 100 kJ mol^{-1} nm^{-1}. This was followed by a two-step equilibration process: initially, a short 1 ns equilibration during which the peptides were pulled toward the membrane surface using a harmonic potential with cylindrical geometry applied to the backbone of the peptide beads, to prevent sampling of peptide in solution. This was followed by an extended 100 ns equilibration following the same equilibration protocol as for the basic planar membrane simulation.

Buckled Bilayer

The system construction started with a flat bilayer comprising 1008 lipids symmetrically distributed across both leaflets. The system was subsequently solvated with approximately 24,000 water beads, and Na and Cl ions were added to attain 0.15 M NaCl concentration. Energy minimization was conducted using the steepest-descent algorithm with a maximal force tolerance of 100 kJ mol^{-1} nm^{-1}. Equilibration proceeded through five stages following the equilibration protocol described above for the planar membrane.

After the initial short equilibration steps, a longer equilibration was carried out with a timestep of 20 fs over a duration of 300 ns, following the same parameters as in the production dynamics of planar membrane.

The membrane was then buckled using PLUMED moving restraints. First, a compressing moving restraint was applied to the box size in the y-direction resulting in a prolonged membrane patch in x-direction. Next, moving restraints were applied in the x-direction while keeping the box size in the y-direction constant, resulting in membrane buckling along x-axis. The extent of bilayer compression was determined by the compression strain γ (Eq. S1), where $L_{x,0}$ is the box length of the planar bilayer in the dimension of compression and $L_{x,i}$ is the box length of the buckled bilayer. A constant value of $\gamma = 0.14$ was used for all membranes.

$$\gamma = \frac{L_{x,0} - L_{x,i}}{L_{x,0}} \tag{S1}$$

The final buckled membranes were then equilibrated for 200 ns, while the buckled shape was preserved by setting the compressibility in x and y directions to 0 bar^{-1}. Water and ions were removed from buckled membrane before adding peptides. One peptide was placed on each membrane leaflet. The system was then re-solvated with ~30,000 water beads, and ions corresponding to the final 0.15 M NaCl concentration were added. The system was energy minimized using the steepest descent algorithm with a maximum force tolerance of 100 kJ mol^{-1}. The snapshot of the prepared system is shown in **??** in the main text.

The equilibration was performed in two steps. First, a short equilibration was performed with a time step of 10 fs and a duration of 1 ns. During this step, backbone beads were restrained by applying a harmonic potential, and later, a 100 ns long equilibration was performed with a time step of 20 fs without any applied biases. In both equilibration steps, the system temperature was kept at 310 K using stochastic velocity-rescaling thermostat (1) and kept at a reference pressure of 1 bar using Berendsen barostat (2) with coupling applied only in z-direction with compressibility set to 3×10^{-4} bar^{-1}. The equilibration steps were followed by production runs of at least 25 µs, using the Parrinello–Rahman barostat (4) with a coupling constant of 12 ps. All other parameters remained consistent with those used during the extended equilibration phase. The initial 2 µs of the simulated trajectories were excluded from the analysis.

Curvature reweighting

Membrane bending is symmetric with respect to the bilayer center. However, as in this work we focus on membrane surface, where there is an imbalance in area of positive and negative curvatures as reported previously (7). We tried to correct for this bias by evaluating the distribution of accessible curvature on membrane surface (as defined by phosphate beads) and used this distribution to reweight the distributions of sampled curvature by peptides using their respective abundance on the membrane surface.

First, we constructed a reference distribution of curvatures present on the membrane surface for membrane-only systems. All the analyzed trajectory frames were aligned with respect to the membrane buckled shape. Second, we used a simple (1D) Fourier series to fit the average membrane buckle, specifically its projection to XZ plane (as opposed to a 2D Fourier series used for frame-by-frame analysis of peptide curvature sensing). We did this to limit the effect of membrane fluctuations, which might make the analysis noisy. When considering the whole trajectory and average membrane shape, using a 1D Fourier series is reasonable as the curvature in the Y-direction (normal to the buckling direction) arises only due to membrane thermal undulations and averages out over time. We then obtained the total profile arc length of the membrane buckle and found 100 equidistant points along the arc length. These points were used to construct a mesh with equidistant Y-points, and at each node, mean curvature was calculated. This analysis was performed for every 100th trajectory frame, enabling us to construct a

distribution of accessible curvature at the membrane surface. A schematic of the procedure used is depicted in Figure S1.

To correct for the non-uniform sampling of curvature imposed by the membrane's buckled geometry, we first computed a reference curvature histogram from every 100th frame of the membrane-only system (for each lipid composition separately). This reference and each peptide's raw mean-curvature values were binned over the same interval of curvatures. For each bin i, we calculated a preliminary weight $w_i = \frac{p_i}{r_i}$, where p_i and r_i are the peptide and reference counts in that bin; bins representing $< 0.1\%$ of either distribution were set to zero to avoid artificial noise. The resulting weighted counts were then normalized, and inverse-transform sampling was used to draw new, bias-corrected curvature values in proportion to w_i. This procedure ensures that all peptides are compared while eliminating artifacts due to uneven sampling resulting from membrane curvature.

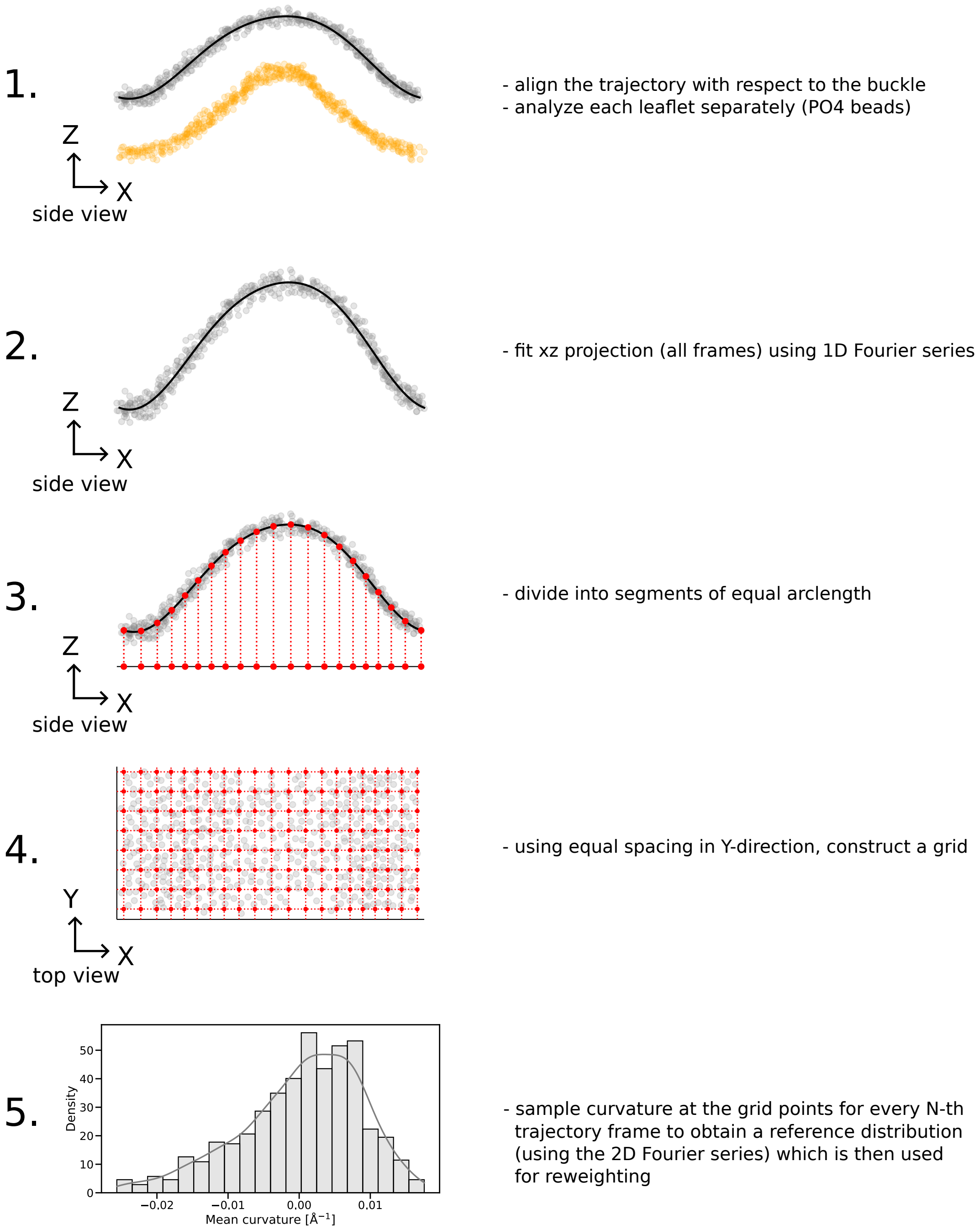

Figure S1: Schematic of the procedure used to obtain reference distributions for the curvature reweighting.

Lateral pressure profiles

To obtain lateral pressure profiles, we extended simulations of planar DOPC, POPC and PAPC membranes, as well as systems containing DOPC membrane and L10 or L15 peptides (by 5 µs. Positions and velocities of all atoms were saved every 500 ps. These trajectories were then post-processed using Gromacs-LS (8), to calculate lateral pressure profiles, $\pi(z)$, along the z-axis using Goetz-Lipowsky force decomposition (9) and 1 Å spacing.

To assess the curvature generating ability of the peptides, we calculated spontaneous monolayer curvature (C_0^m) of pure membrane and of membrane with peptides from planar membrane systems we used relation between the first moment of the lateral pressure profile and bending moment, $\kappa_m C_0^m$ (Eq. S2). In order to calculate C_0^m, knowledge of monolayer bending modulus, κ_m is necessary. Here, we used value of $5.9 \pm 0.2 \times 10^{-20}$J (from (10)).

$$\kappa_m C_0^m = \int_0^\infty z\,\pi(z)\,\mathrm{d}z \tag{S2}$$

Table S1: Spontaneous monolayer curvature (C_0^m) for DOPC membrane and DOPC membrane with peptides L10 and L15. For calculation of spontaneous curvature, monolayer bending modulus for DOPC of $5.9 \pm 0.2 \times 10^{-20}$J was used, based on (10). The error was determined from asymmetry of the lateral pressure profile.

Peptide	C_0^m [nm^{-1}]
DOPC	-0.168 ± 0.006
+L10	-0.167 ± 0.006
+L15	-0.178 ± 0.006

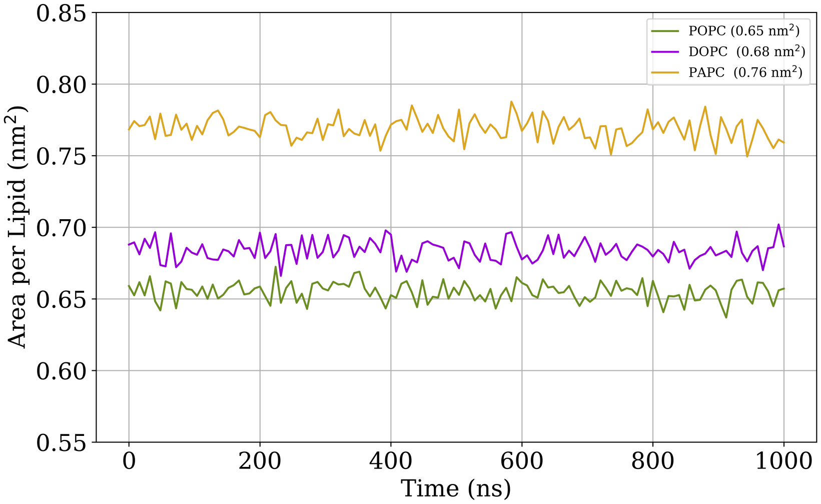

Figure S2: Area per lipid of POPC, DOPC, and PAPC planar membranes without peptides; the mean values are shown in the legend.

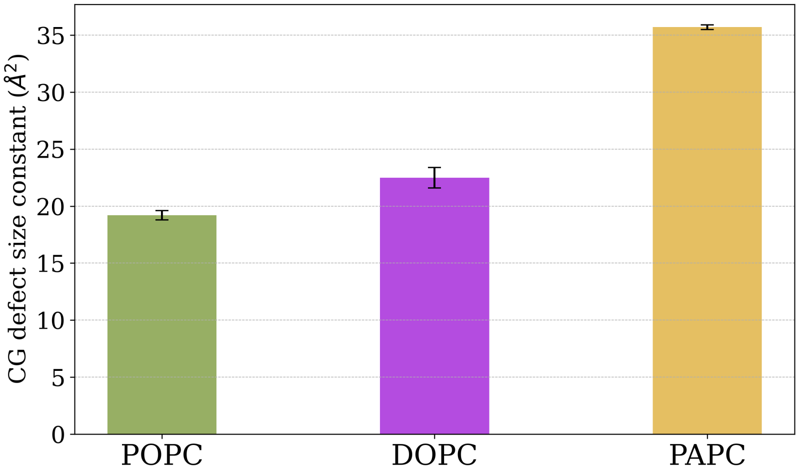

Figure S3: Area of lipid packing defects for the membranes of POPC, DOPC, and PAPC planar membranes using Martini model.

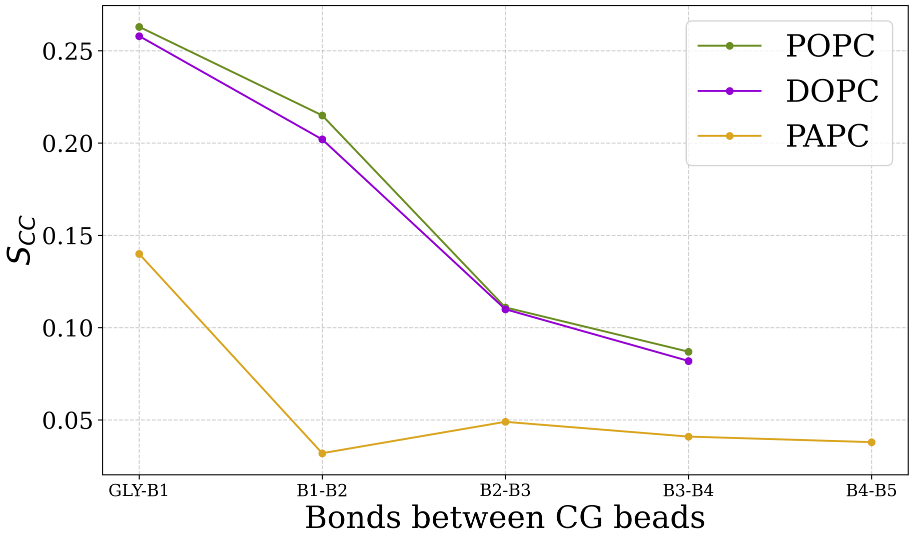

(a) S_{CC} Order parameters for sn-1 chain of POPC, DOPC and PAPC planar membranes.

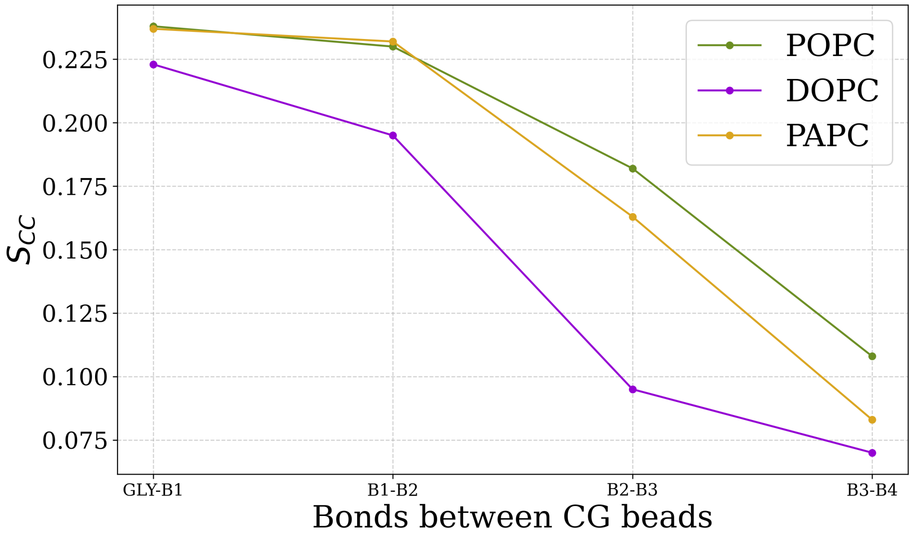

(b) S_{CC} Order parameters for sn-2 chain of POPC, DOPC and PAPC planar membranes.

Figure S4: S_{CC} Order parameters for sn-1 and sn-2 acyl chains of the POPC, DOPC, and PAPC planar membranes. GLY in the x-axis represent the glycerol bead and B1, B2, B3, B4 and B5 represent the beads along the acyl chains. a) Order paremeters of sn-1 acyl chain. B4-B5 beads is only valid for PAPC membrane b) Order Parameters of sn-2 acyl chain.

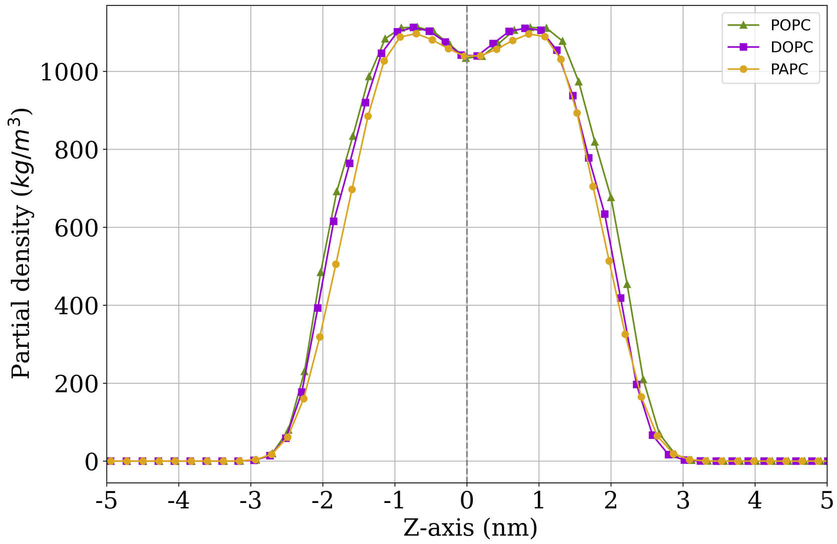

Figure S5: Partial density profiles of POPC, DOPC, and PAPC planar membranes.

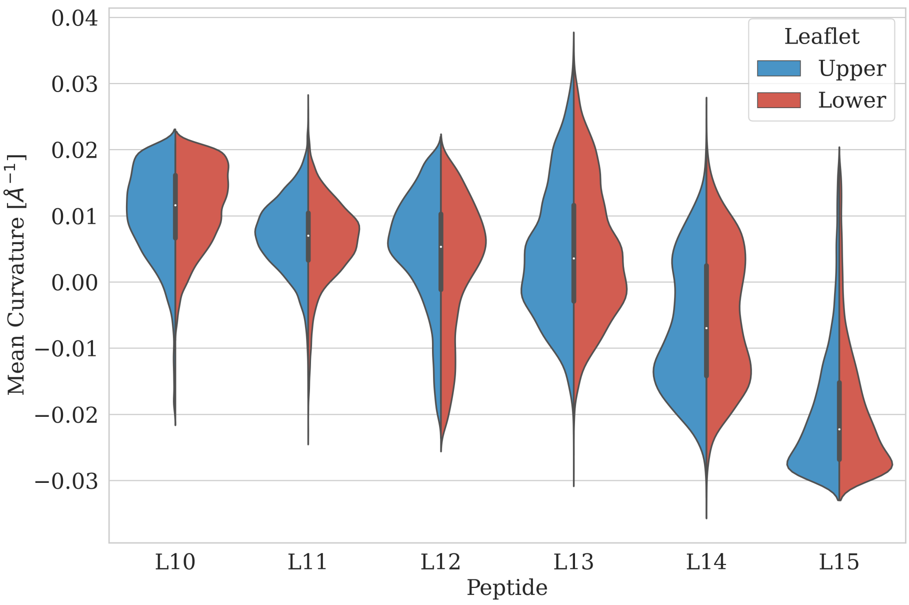

Figure S6: Reweighted mean curvature distribution for L10-L15 peptide on POPC buckled membrane.

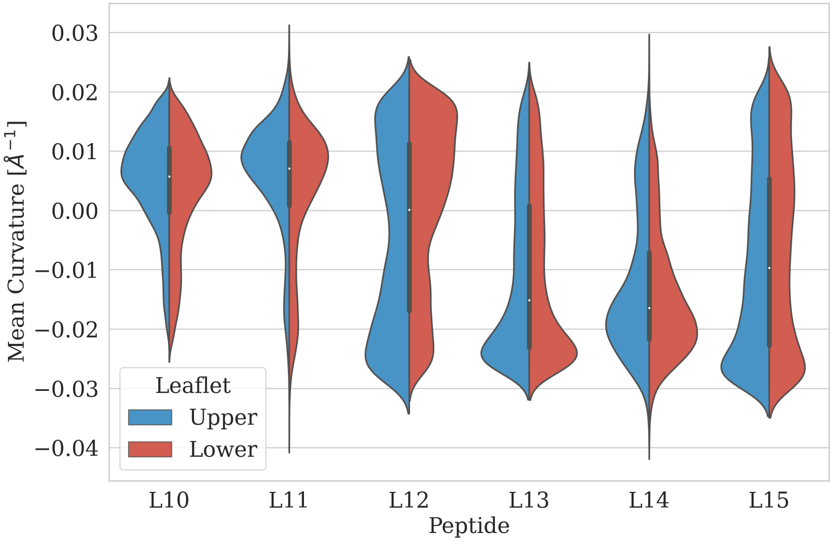

Figure S7: Reweighted mean curvature distribution for L10-L15 peptide on DOPC buckled membrane.

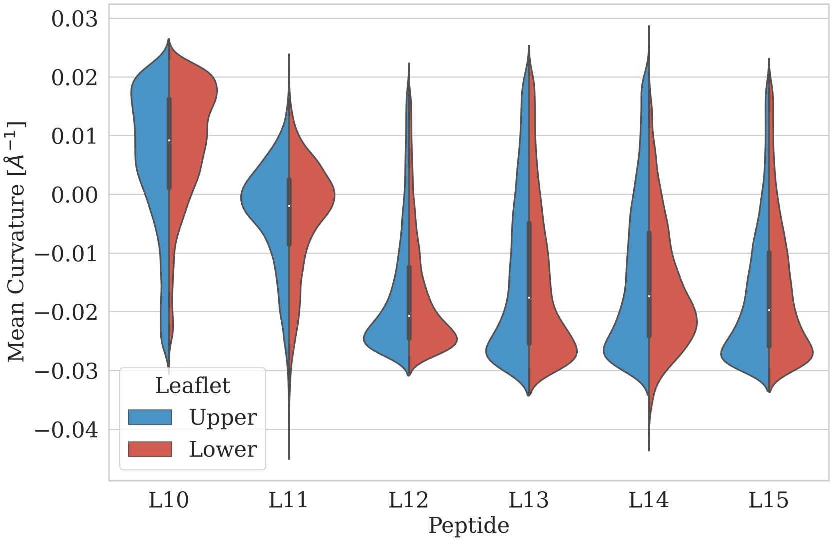

Figure S8: Reweighted mean curvature distribution for L10-L15 peptide on PAPC buckled membrane.

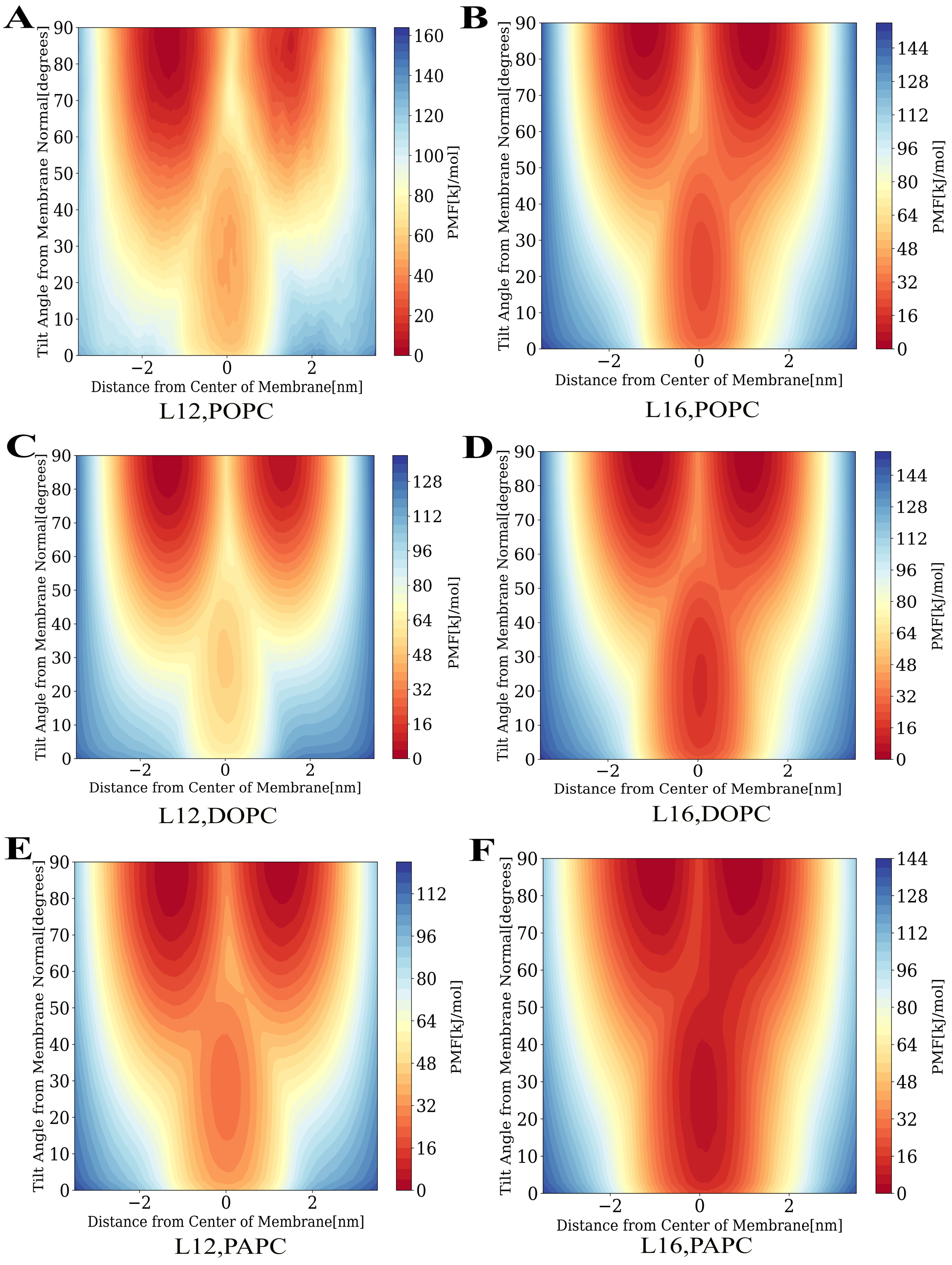

Figure S9: 2D Free energy profiles for the L12 and L16 peptides on POPC, DOPC, and PAPC planar membranes.

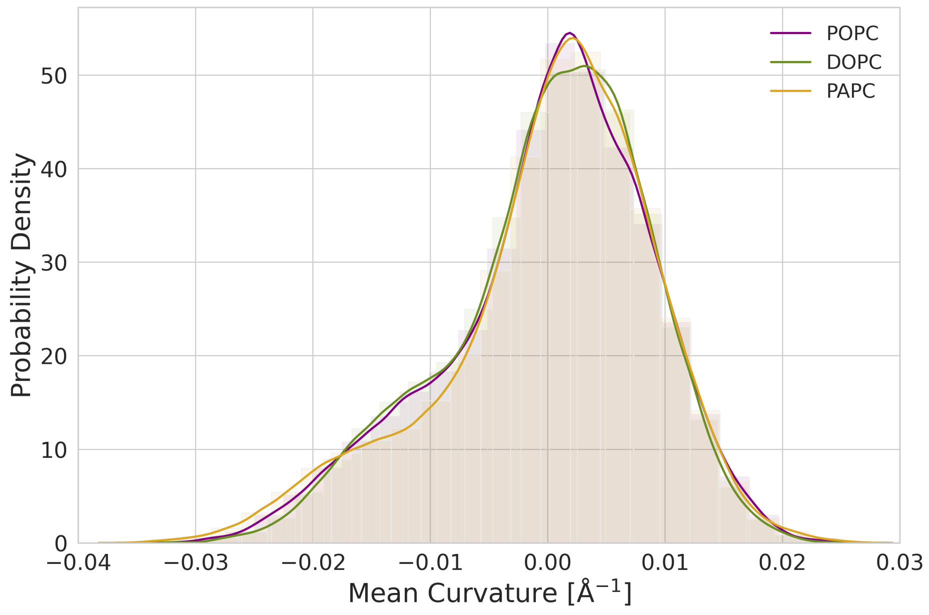

Figure S10: Accessible curvatures on POPC, DOPC, and PAPC buckled membranes.

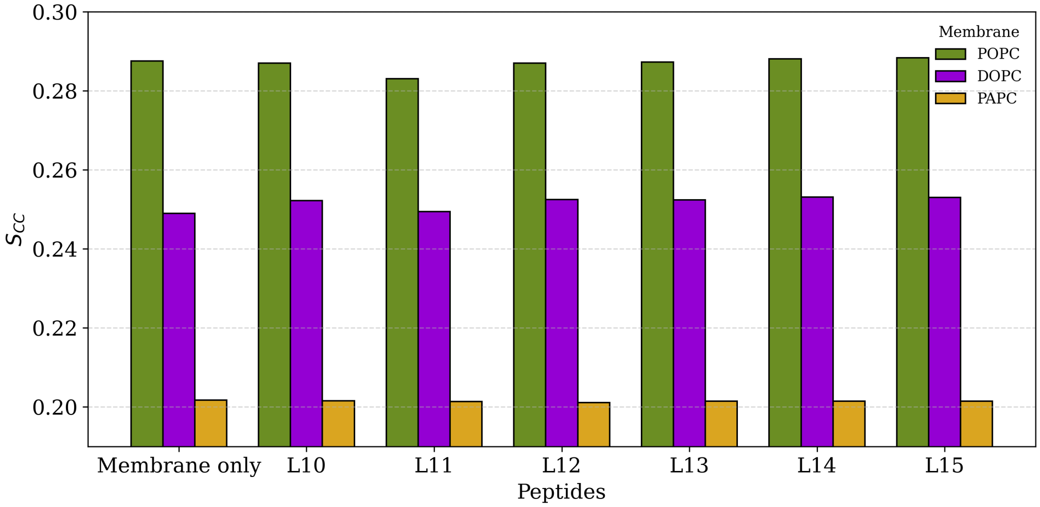

Figure S11: Average order parameters for POPC, DOPC and PAPC membrane within 2.5 nm of radius around the the peptides in the flat-membrane simulations.

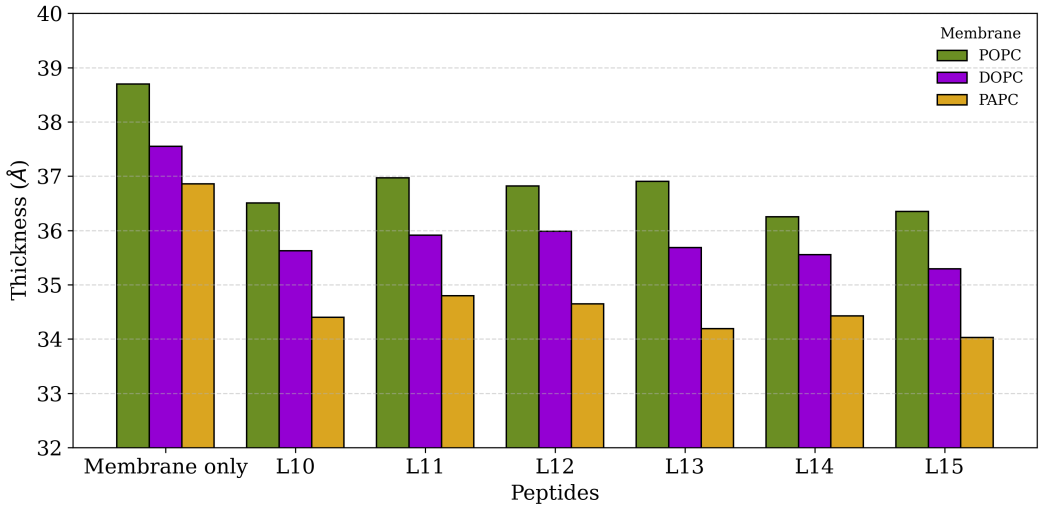

Figure S12: Average membrane thickness for POPC, DOPC and PAPC membrane within 2.5 nm around the peptide in the flat-membrane simulations.

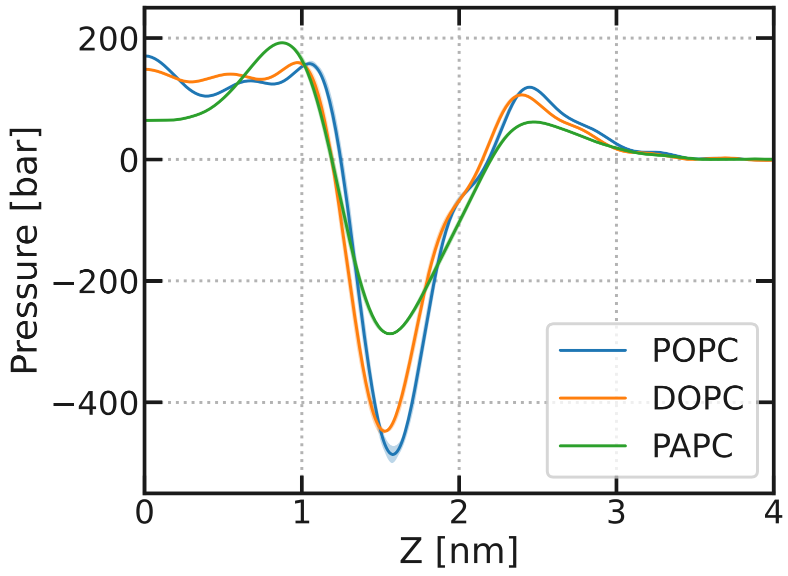

Figure S13: Lateral pressure profiles of POPC, DOPC, and PAPC membranes. As the profiles were symmetrized, only one leaflet is shown.

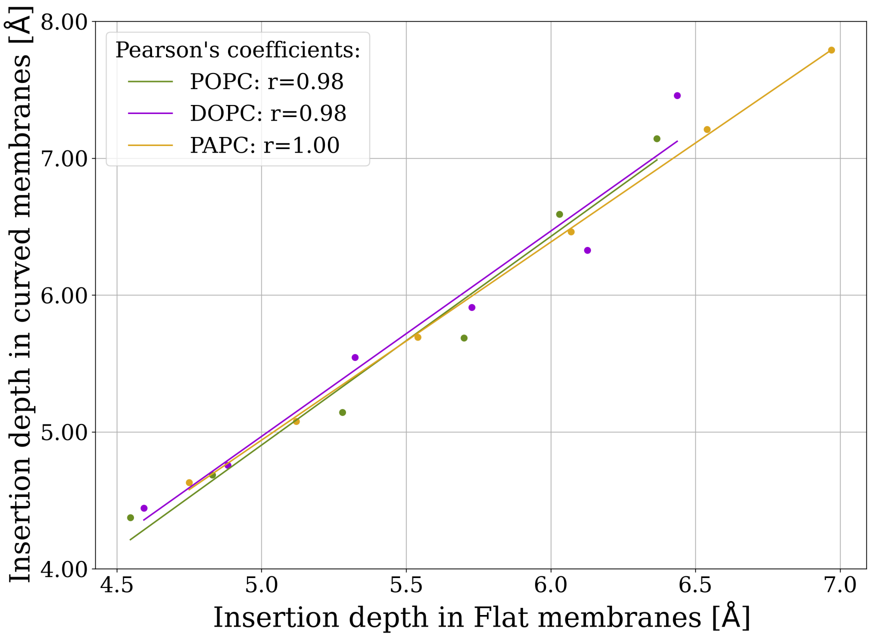

Figure S14: Correlation plot of peptide insertion depth in curved membrane against its insertion depth in planar bilayers.

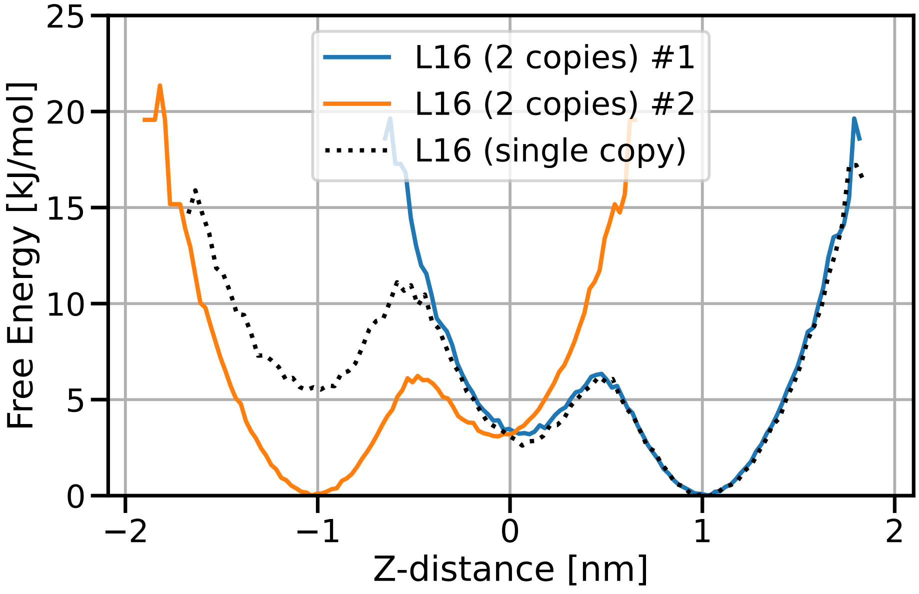

(a) The free energy profiles of peptide reorientation obtained from unbiased simulations on PAPC membrane with either single L16 peptide or one peptide copy on each leaflet (2 copies in total).

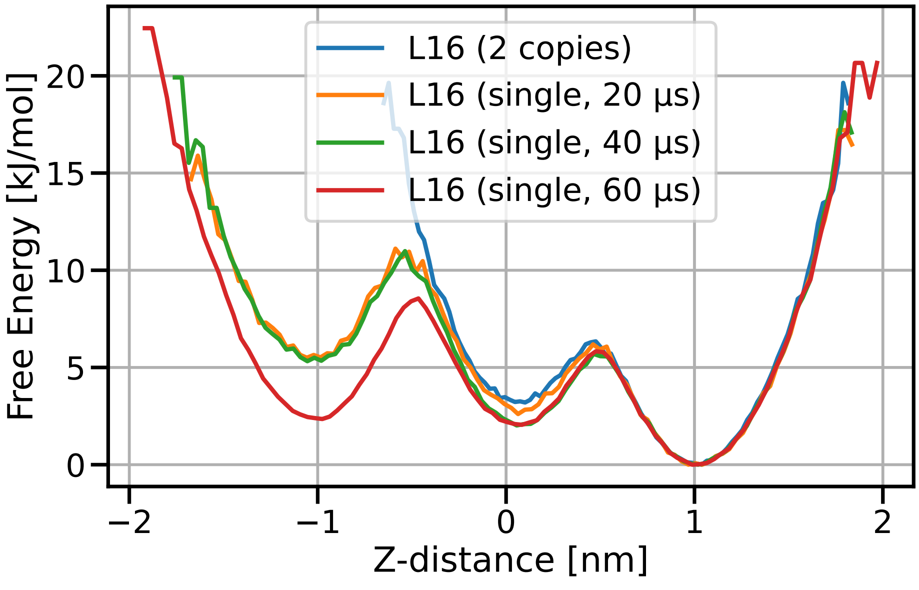

(b) The free energy profiles obtained from prolonged simulation of the system with a single L16 copy demonstrate the asymmetry of the unbiased simulation profile originated from the insufficient sampling within the used time scale.

Figure S15: Comparison of free energy profiles obtained from unbiased simulations containing two or a single copy of the L16 peptide.

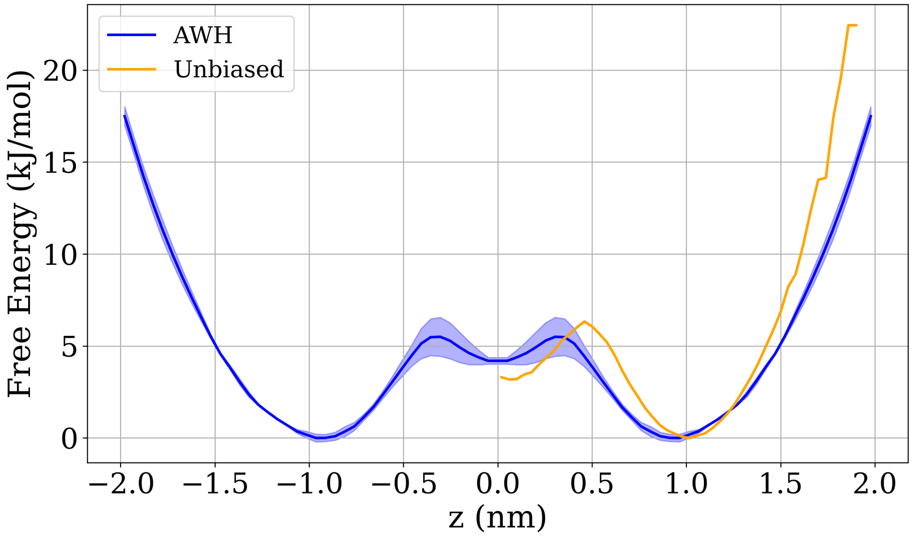

Figure S16: The plot shows the comparision of the free energy from unbiased simulations and AWH simulations for L16 peptide on PAPC membrane.

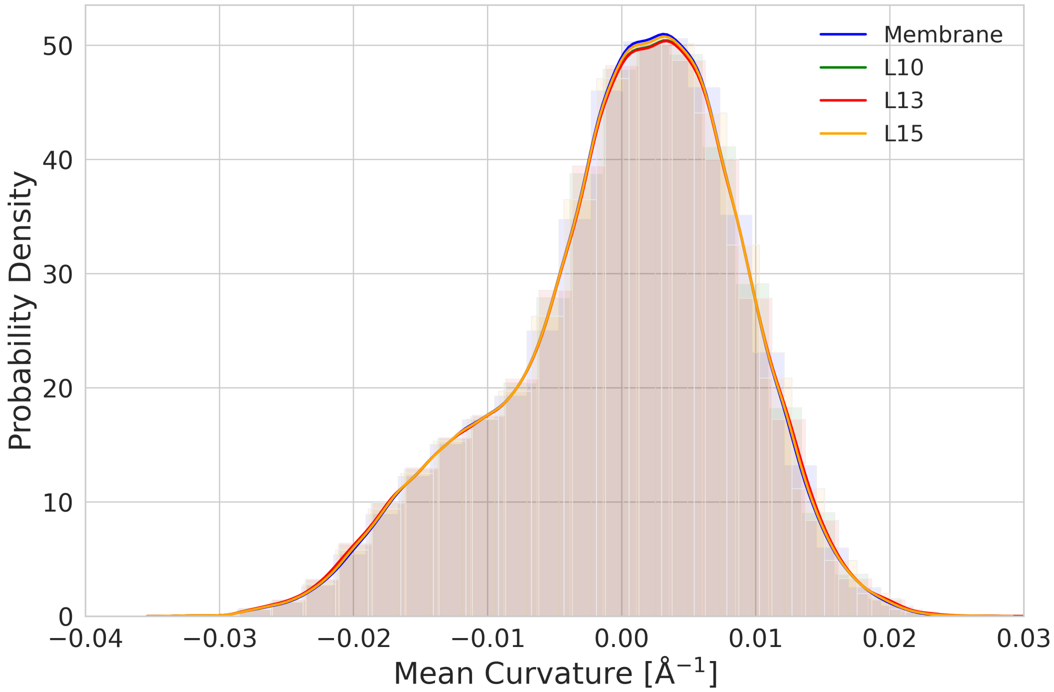

Figure S17: The accessible curvatures for L10, L12 and L15 peptide on DOPC membrane.

REFERENCES

1. Giovanni Bussi, Davide Donadio, and Michele Parrinello. “Canonical sampling through velocity rescaling”. In: *The Journal of chemical physics* 126.1 (2007).
2. Herman JC Berendsen et al. “Molecular dynamics with coupling to an external bath”. In: *The Journal of chemical physics* 81.8 (1984), pp. 3684–3690.
3. Djurre H De Jong et al. “Martini straight: Boosting performance using a shorter cutoff and GPUs”. In: *Computer Physics Communications* 199 (2016), pp. 1–7.
4. Michele Parrinello and Aneesur Rahman. “Polymorphic transitions in single crystals: A new molecular dynamics method”. In: *Journal of Applied physics* 52.12 (1981), pp. 7182–7190.
5. Romain Gautier et al. “PackMem: a versatile tool to compute and visualize interfacial packing defects in lipid bilayers”. In: *Biophysical journal* 115.3 (2018), pp. 436–444.
6. Ladislav Bartoš, Peter Pajtinka, and Robert Vácha. “gorder: Comprehensive tool for calculating lipid order parameters from molecular simulations”. In: *SoftwareX* 31 (2025), p. 102254. ISSN: 2352-7110. DOI: https://doi.org/10.1016/j.softx.2025.102254.
7. Kevin J Boyd, Nathan N Alder, and Eric R May. “Buckling under pressure: curvature-based lipid segregation and stability modulation in cardiolipin-containing bilayers”. In: *Langmuir* 33.27 (2017), pp. 6937–6946.
8. Juan M Vanegas, Alejandro Torres-Sánchez, and Marino Arroyo. “Importance of force decomposition for local stress calculations in biomembrane molecular simulations”. In: *Journal of chemical theory and computation* 10.2 (2014), pp. 691–702.
9. Rüdiger Goetz and Reinhard Lipowsky. “Computer simulations of bilayer membranes: self-assembly and interfacial tension”. In: *The Journal of chemical physics* 108.17 (1998), pp. 7397–7409.
10. Richard M Venable, Frank LH Brown, and Richard W Pastor. “Mechanical properties of lipid bilayers from molecular dynamics simulation”. In: *Chemistry and physics of lipids* 192 (2015), pp. 60–74.
