# Supplementary material for: Helical sensors of membrane saturation: Changes in orientation and curvature preference
Source: Biophys J. 2025 Oct 3;124(23):4087–95. doi: 10.1016/j.bpj.2025.09.042 (PMC12709426; doi:10.1016/j.bpj.2025.09.042)
Supplement: Document S2. Article plus supporting material [file mmc2.pdf]

Biophysical Journal
Article

Biophysical Society

Helical sensors of membrane saturation: Changes in orientation and curvature preference

Sushmita Pal,[1,2] Peter Pajtinka,[1,2] Matti Javanainen,[4,5] and Robert Vácha[1,2,3,*]
[1]CEITEC – Central European Institute of Technology, Masaryk University, Brno, Czech Republic; [2]National Centre for Biomolecular Research, Faculty of Science, Masaryk University, Brno, Czech Republic; [3]Department of Condensed Matter Physics, Faculty of Science, Masaryk University, Brno, Czech Republic; [4]Unit of Physics, Tampere University, Tampere, Finland; and [5]Institute of Biotechnology, University of Helsinki, Helsinki, Finland

ABSTRACT The degree of unsaturation in lipids, which refers to the number of double bonds in their acyl chains, influences properties such as fluidity and lipid packing. However, it is not well understood how the unsaturation affects the ability of peptides to sense membrane curvature. In our study, we compared membranes with varying levels of unsaturation: monounsaturated POPC; bis-unsaturated DOPC; and polyunsaturated PAPC. We investigated how these membranes interact with peptides of varying hydrophobicity. Using coarse-grained molecular dynamics simulations, we found that increasing unsaturation leads to deeper peptide insertion into the lipid bilayer, which correlates with a shift in curvature preference toward more negative values. We demonstrate that specific peptides preferentially localize on the positively curved regions in saturated membranes but shift preference to negatively curved regions in unsaturated membranes, thereby functioning as sensors of membrane unsaturation. In addition, polyunsaturated lipids facilitate the reorientation of peptides from a membrane-adsorbed state to a transmembrane state. These findings may play a role in biological processes such as vesicle formation, membrane fusion, and protein sorting and highlight the adaptability of peptides to different lipid compositions in membranes.

SIGNIFICANCE Unsaturated lipids contain one or more double bonds in their acyl chains. These unsaturated lipids are abundant in cellular organelles such as the endoplasmic reticulum and in specific tissues including human heart and brain. However, it remains unclear how the presence of unsaturated lipids affects the function of membrane proteins. This study demonstrates that a higher degree of membrane unsaturation results in deeper insertion of amphiphilic peptides, shifting the sensing of membrane curvature to negative values. Moreover, stabilization of transmembrane state in an unsaturated membrane can lead to reorientation of α-helices depending on the membrane unsaturation level, providing a possibility for the regulation of protein localization and function.

INTRODUCTION

The interaction of peripheral proteins with membranes is governed by a complex interplay of factors including protein sequence, lipid composition, solvent environment (1–3), physical constraints such as curvature, spatial organization of membrane proteins, and the lipid phase behavior (4–6). These interactions are critical for the structural integrity and functional dynamics of cellular membranes.

As membrane-bound proteins progress through the secretory pathway—from the endoplasmic reticulum (ER) to the Golgi apparatus and ultimately to the plasma membrane—they encounter distinctly different lipid environments (3,7–9). The ER is a complex system of interconnected membrane discs and cylinders and is rich in unsaturated lipids, which possess one or more double bonds in their acyl chains. In contrast, the relatively flat plasma membrane has a higher concentration of saturated lipids (10,11). These variations in lipid composition not only influence the physical properties of the membrane, such as water permeability and fluidity, but are also crucial for the appropriate sorting and function of membrane proteins and peptides (12). Furthermore, these membrane-bound proteins can sense and adapt to the changing lipid environments by exhibiting a differential topological orientation within the membrane (13).

Curvature-sensing proteins can preferentially localize to or interact with membranes of specific curvature. They

Submitted April 10, 2025, and accepted for publication September 26, 2025.
*Correspondence: robert.vacha@muni.cz
Editor: Edward Lyman.
https://doi.org/10.1016/j.bpj.2025.09.042
© 2025 The Author(s). Published by Elsevier Inc. on behalf of Biophysical Society.
This is an open access article under the CC BY license (http://creativecommons.org/licenses/by/4.0/).

are able to respond to changes in membrane curvature and regulate a variety of cellular processes, including vesicle trafficking and endocytosis (5,14). The ability to sense curvature is attributable to their curvature-sensing motifs, mainly BAR (Bin/Amphyphisin/Rvs) domains or amphipathic helices (AHs) (14,15). The BAR domain forms an arched structure that enables it to discriminate membrane curvature (16).

AHs, in contrast to BAR domains, do not contain intrinsically curved surfaces, which would explain their curvature preference. Instead, their curvature-sensing mechanism was interpreted as binding to lipid-packing defects (17,18), i.e., imperfections in the arrangement of lipid molecules within the bilayer. These defects are enriched in membrane regions of positive curvature where packing of lipid headgroups is looser (18). AHs, such as the ALPS (amphipathic lipid packing sensor) peptide, can thus effectively sense positive membrane curvature. Nevertheless, they could sense also different regions as lipid packing defects can also arise from the presence of tension or nonlamellar lipids (19).

Campelo and Kozlov proposed a distinct mechanism for curvature sensing of AHs, emphasizing the role of internal membrane stress (20). Due to membrane bending, significant internal stresses arise within the lipid headgroups in regions of positive curvature. These stresses lead to packing defects connecting the two proposed mechanisms. However, membrane bending also alters intramembrane stresses beyond the headgroup region (21), potentially providing additional cues for peptide interaction, especially in regions with negative curvature, where lipid defects are reduced. This mechanism thus extends the concept of curvature sensing to negatively curved membrane regions. Recently, AHs able to recognize negative membrane curvature were identified (22).

Membrane proteins play vital roles in cellular functions and are generally considered to have stable transmembrane orientations. Yet, recent findings increasingly support that these transmembrane helices are topologically malleable and can undergo dynamic reorientation with shifts in the lipid milieu (13,23). Experimentally investigating these subtle yet crucial changes at physiologically relevant timescales is challenging. Therefore, molecular dynamics simulations are ideal to provide detailed insights into the energetics and mechanisms that underlie the conformational flexibility of membrane proteins.

In this study, the primary objective was to elucidate the impact of lipid unsaturation on peptide-membrane interactions. To this end, coarse-grained simulations were employed to analyze the effects of varying lipid bilayer unsaturation on peptide insertion and subsequent reorientation into a transmembrane state. To further investigate these mechanisms, free energy calculations were conducted to determine peptide orientation preferences. Moreover, the localization and behavior of these peptides on curved bilayers were analyzed, with a focus on how varying levels of lipid unsaturation and membrane curvature collectively influence peptide sorting. The findings of this study highlight the dynamic interplay between peptide properties and membrane composition.

MATERIALS AND METHODS

All-atom structures of the 21-residue-long peptides, composed solely of leucine and serine (see Table 1), were generated using MODELLER (24) version 9.11. They were constructed as fully α-helical and subsequently converted to a coarse-grained representation using the martinize.py script, version 2.6, with an enforced α-helical secondary structure (25). The investigated peptides have systematically varied size of hydrophobic patches to evaluate the impact of hydrophobicity on their interaction with membranes of different unsaturation levels. The scale of hydrophobicity is derived from N-acetyl-amino-acid amides' partitioning between water and octanol (26).

A total of three membrane lipid compositions were examined, each exhibiting progressively higher degree of unsaturation. Specifically, these membranes consisted of phosphatidylcholine (PC) lipids with PO (palmitoyl-oleoyl, 16:0-18:1), DO (dioleoyl, 18:1-18:1), or PA (palmitoyl-arachidonoyl, 16:0-20:4) acyl chains. All initial membrane structures were generated using CHARMM-GUI web server (27,28), and subsequent simulations were performed using GROMACS (29) version 2021.4 with the PLUMED (30) plugin, in combination with coarse-grained Martini 2.2 force field (25,31), which was successfully applied to study curvature sensing and sorting of proteins and lipids alike (18,22,32).

In this study, we have employed two types of membrane systems: buckled and planar membranes. Both will be introduced separately.

Detailed simulation protocols for the preparation of all systems are provided in the supporting material.

Planar membrane

Insertion depth

The system with planar membrane comprises 184 lipid molecules (92 per leaflet) and two peptide copies, with one copy placed on each leaflet in the headgroup region with parallel orientation to the membrane plane. Membrane systems were prepared separately for POPC, DOPC, and PAPC lipid types with peptides L10 through L15. Molecular dynamics simulations were performed for 40 μs per system with three independent replicates.

During the analysis, we quantified the depth of peptide insertion, which we defined as the z-distance between the center of mass (COM) of the peptide and phosphate beads of the corresponding leaflet within a cylindrical selection with radius of 1.0 nm. The simulations were performed for 40 μs, and the simulation configurations were saved after every 400 ps, yielding a total of 100,000 configurations. The z-distance was then

TABLE 1 Summary of the peptide labels used in this work, their respective amino acid sequences, and mean hydrophobicity

Peptide	Sequence	Hydrophobicity
L10	LSSLLSLLSSLLSSLSSLLSS	0.789
L11	LSSLLSLLSSLLSLLSSLLSS	0.871
L12	LSSLLSLLSSLLSLLSSLLSL	0.954
L13	LSLLLSLLSSLLSLLSSLLSL	1.037
L14	LSLLLSLLSLLLSLLSSLLSL	1.120
L15	LSLLLSLLSLLLSLLSLLLSL	1.203
L16	LSLLLLLLSLLLSLLSLLLSL	1.286

calculated for all configurations, and the mean value was reported as the insertion depth.

Free energy calculation

Planar membrane systems consisting of 400 lipids each were constructed for POPC, DOPC, and PAPC lipid types, and free energy calculations were performed for peptides L12 and L16. Only a single peptide copy, placed on the upper leaflet of the membrane, was used in these simulations. Accelerated weight histogram (AWH) method was employed to calculate the potential of mean force (PMF) to study the orientation preference of the peptide within the membrane (33). AWH is an enhanced sampling method that adaptively applies a bias along selected reaction coordinates to efficiently obtain the PMF. Unlike umbrella sampling, AWH does not require tuning of parameters based on the shape of the free energy landscape, making it effective across both flat and steep regions with a single set of parameters.

Two collective variables (CVs) were used: 1) the distance of the COM of the peptide to the COM of the membrane (CV1) and 2) the angle between the peptide and the z-axis (parallel to the membrane normal, CV2) were used to calculate the free energy profiles. The input force constants were set to 1000 kJ mol^{-1} nm^{-2}. The default setup of convolution of Gaussians produced by harmonic umbrellas was used as the AWH potential. The simulations were performed for 25 μs. The PMFs were extracted from the AWH method as implemented in GROMACS tool `gmx awh`. The obtained free energy profiles were symmetrized for the peptide sampling the upper and lower leaflet, and the errors were estimated from these two independent data sets. The one-dimensional PMF along the CV1 was obtained by Boltzmann averaging as

$$\Delta G(\mathrm{CV1}) = -RT \ln\left(\frac{\int e^{-\beta \Delta G(\mathrm{CV1},\mathrm{CV2})}\ \mathrm{dCV2}}{C}\right), \quad (1)$$

where R is the universal gas constant, and C with units of length ensures a dimensionless argument of the logarithm. The obtained one-dimensional free energy profile was then vertically shifted so that $\Delta G_{min} = 0$.

Buckled membrane

The simulations on buckled bilayers were performed for POPC, DOPC, and PAPC membranes for peptides L10 through L15 for at least 25 μs. A snapshot of the prepared system is shown in Fig. 1.

Trajectories were analyzed utilizing the in-house modified version of the MemCurv Python package, developed by Bhaskara et al. (34). In brief, the method is based on a Monge gauge (35), where the membrane surface is described by a single function, in this case, the height function, obtained by fitting positions of phosphate beads. For this purpose, a two-dimensional (2D) Fourier series was used together with least squares optimization. Subsequently, the obtained membrane surface enabled the calculation of principal curvatures and corresponding mean and Gaussian curvatures at a given point. The analysis was performed separately for each system configuration in the trajectory. To remove the uneven sampling resulting from the underlying geometry of membrane buckle, the distributions of sampled curvature were reweighted using the distribution of accessible curvature at membrane surface. Further details are provided in Supplementary Methods.

RESULTS

The biophysical properties of the studied membranes including area per lipid, order parameters, lipid packing defects, and partial density profiles are displayed in Figs. S2, S3, S4, and S5, respectively. An increased level of unsaturation results in a larger area per lipid, as evidenced by calculated values of (65 ± 1) Å^2, (68 ± 1) Å^2, and (76 ± 1) Å^2 for POPC, DOPC, and PAPC membranes, respectively (Fig. S2). Similarly, lipid-packing defects were also affected, and their incidence increased with higher unsaturation. The corresponding defect area constants for POPC, DOPC, and PAPC are (19.2 ± 0.4) Å^2, (22.5 ± 0.9) Å^2, and (35.7 ± 0.2) Å^2, respectively. Fig. S3 illustrates the positive correlation between lipid unsaturation and the incidence of packing defects. These findings are in agreement with previous experimental and computational studies (36–40).

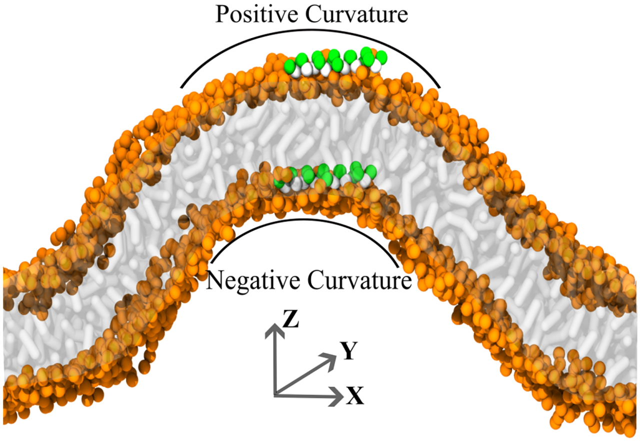

FIGURE 1 A representative snapshot of the buckled bilayer system. One peptide was placed on each membrane leaflet, one in the region of positive and the other in the region of negative membrane curvature. Throughout the simulation, the peptides were able to diffuse freely.

Membrane unsaturation was also found to have an impact on the insertion depth of the studied peptides (Fig. 2 *D*). The peptides inserted deeper into the membrane as the level of unsaturation increased; see Fig. 2. Higher peptide hydrophobicity (by mutating serine to leucine residues, raising their count from 10 in L10 to 15 in L15) also resulted in a deeper peptide insertion Fig. 2 *D*). This effect was more pronounced on polyunsaturated PAPC membrane, compared with both POPC ($p < 0.05$) and DOPC ($p < 0.05$), whereas POPC and DOPC showed equal trends of insertion depth ($p = 0.67$). The significance of the different slopes was estimated with two-sample t-tests.

We also investigated the local effect of the peptides on the membrane properties, specifically its thickness (Fig. S11) and S_{CC} order parameter (Fig. S12); for details of the methods, see the Supporting Material. We observed that the local lipid order parameters around the peptides did not vary significantly. However, the local membrane thickness was consistently lower around peptides, indicating a thinning effect on the tested membranes.

With increasing hydrophobicity of peptides, there is an increasing chance that the peptides would reorient and become transmembrane. Indeed, we observed a spontaneous transmembrane reorientation of hydrophobic peptides in lipid membranes with different degrees of unsaturation in

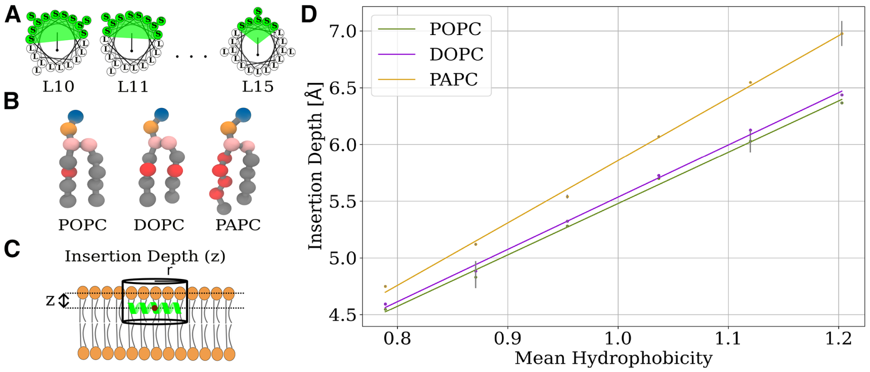

FIGURE 2 The effect of peptide hydrophobicity on the depth of insertion within the membrane with varying degrees of unsaturation, namely POPC, DOPC, and PAPC. (*A*) Helical wheel diagrams of studied peptides with increasing size of hydrophobic patch. (*B*) Employed lipids depicted in Martini model. The gray beads represent the hydrophobic acyl chains, whereas the red beads represent the unsaturated beads in the tails. The orange and blue beads represent the phosphate and choline beads, respectively. (*C*) The depth of peptide insertion in the membrane is defined as the z-distance between the peptide center of mass and lipid phosphates within a cylindrical selection with radius (r) of 1.0 nm. (*D*) The depth of peptide insertion in POPC, DOPC, and PAPC membranes as a function of peptide mean hydrophobicity. The error bars represent the standard deviation calculated from three independent simulations.

our unbiased molecular dynamics simulations. The propensity of the peptides to go transmembrane was observed primarily on polyunsaturated membranes of PAPC for the L16 peptide (Fig. 3). The peptide repeatedly sampled the adsorbed and transmembrane states, due to a low energy barrier between them. Since L16 spontaneously reorients itself to a transmembrane state, the depth of insertion is not addressed for this peptide in Fig. 2.

The transmembrane orientation of peptides in our unbiased simulations could represent the metastable state or the local minima, and hence, to get insights into the energetics of the reorientation, we performed free energy calculations on two model peptides: a moderately hydrophobic peptide, L12, and a highly hydrophobic peptide, L16. We used the AWH method to calculate free energy landscapes along two CVs to distinguish among structurally distinct peptide configurations that could overlap when described solely by one collective variable. These CVs were 1) the COM distance between the peptide and the membrane (CV1) and 2) the tilt angle of the peptide relative to the z-axis (CV2). Fig. S9 shows the 2D free energy map, where the abscissa represents CV1, and the ordinate represents CV2.

Fig. 4 shows the free energy profiles of translocation as a function of the distance between the peptide and the membrane COM. These profiles were derived from Boltzmann averaged data across CV1. There are three states: 1) the peptide adsorbed on the lower leaflet, 2) the peptide in a transmembrane orientation, and 3) the peptide adsorbed on the upper leaflet.

All 2D free energy profiles for peptides L12 and L16 on POPC, DOPC, and PAPC membranes are shown in Fig. S9. The profiles indicate that the free energy barrier for reorientation was lower for hydrophobic peptide L16, and this effect was more pronounced in PAPC. Here, we defined $\Delta\Delta G$ as the difference in free energy between the adsorbed state and the transmembrane state. This metric reflects the relative probability of a peptide being in the adsorbed state compared with the transmembrane state. The free energy differences obtained using the AWH method are summarized in Table 2.

Our analysis demonstrates that for the amphipathic peptide L12 in the monounsaturated POPC membrane, $\Delta\Delta G$ is 52.7 $\pm$ 1.3 kJ/mol, indicating a pronounced preference for the adsorbed state over the transmembrane state. The hydrophobic peptide L16 exhibits a markedly lower $\Delta\Delta G$ of 19.8 $\pm$ 0.6 kJ/mol in the same membrane. Note that in the polyunsaturated PAPC membrane, the free energy barrier for reorientation of L16 was decreased, with $\Delta\Delta G$ to only 4.2 $\pm$ 0.2 kJ/mol. This result is in accordance with the findings of unbiased simulations, in which the L16 peptide was found to spontaneously reorient from the peripheral state to the transmembrane state in the PAPC membrane (even when only a single peptide copy was present on the membrane). This reorientation occurred due to a very low free energy barrier for the process and could be driven by mere thermal fluctuations at physiological conditions (Fig. S15). To validate the free energies obtained from the enhanced sampling AWH method, we also computed free energy profiles from unbiased simulations using Boltzmann inversion of the density distribution, as shown in Fig. S16. Both methods yielded similar free energy barriers, demonstrating consistency between the AWH and unbiased approaches.

In addition to the understanding of the energetics of peptides and their dynamic behavior in varying lipid environments, we evaluated the peptide curvature preference in relation to their hydrophobicity and the lipid composition of the membrane. The probability distribution of the sampled curvatures was reweighted with accessible membrane curvatures to capture the curvature preferences of the studied peptides (see Fig. S10). The convergence of the results was validated by observing a consistent curvature distribution between the two peptides on the upper and lower leaflets of the buckled bilayer. See the Supplementary Methods section for more details and the Supporting Material for all the obtained curvature distributions (Figs. S6, S7, and S8). The effect of peptides on membrane curvature was negligible under the concentrations used in this study (Table S1; Fig. S17).

Due to their distinct physicochemical properties, we focused on three peptides, L10, L12, and L15 (Fig. 5).

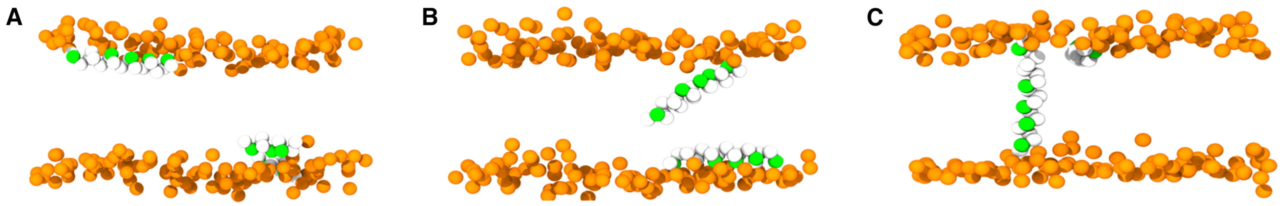

FIGURE 3 The snapshots from an unbiased coarse-grained simulation showing spontaneous reorientation of the peptide in the membrane. (*A*) Peptide in adsorbed state, (*B*) in inserted state, and lastly, (*C*) peptide in transmembrane state. The orange beads represent the lipid headgroups. The white and green beads represent the peptide beads of leucines and serines, respectively. The acyl chains are not shown for clarity.

Peptide L10 is hydrophilic and serine-rich, L15 is distinctly hydrophobic, and L12, with its amphipathic nature, mediates between them in hydrophilicity. Fig. 5 depicts the reweighted sampled curvatures on the POPC, DOPC, and PAPC membranes for these peptides. The reweighting was done due to known imbalance in area of positive and negative curvature at membrane surface (41). The curvature preferences of the peptides were found to correlate with their depth of insertion into the planar membrane, regardless of the lipid type (Fig. 6). The L10 peptide, which is highly hydrophilic, interacted predominantly with the headgroups of the membrane and preferentially localized to regions of positive curvature. As the peptides penetrated deeper into the membrane, they exhibited tendency to sample regions of negative curvature.

In the case of the L15 peptide, its higher hydrophobicity enabled a deeper insertion into the membrane compared with the L10 and L12 peptides, resulting in the sampling of negative curvatures across all membrane types (POPC, DOPC, and PAPC). In contrast, the L12 peptide, with a more balanced amphipathic nature, exhibited different curvature preferences that were dependent upon the membrane composition. Specifically, the L12 peptide localized to positive curvatures on POPC, whereas it localized to negative curvatures on PAPC and exhibited no clear curvature preference on DOPC membrane (Fig. 5). Consequently, we hypothesize that peptides with properties similar to the L12 peptide would be capable of sensing membrane unsaturation and partition themselves into membrane regions with different curvature depending on lipid composition.

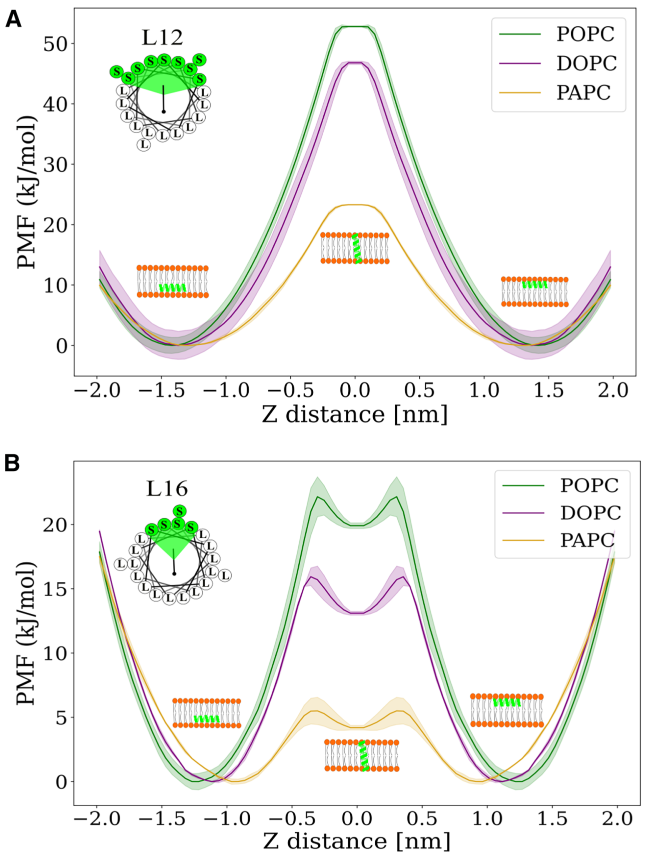

FIGURE 4 The free energy profiles for the peptide reorientation in the membranes POPC, DOPC, and PAPC. The peptide L12 shows consistently higher energetic barrier than L16 to reorient itself into the transmembrane state as expected. Note that L16 has a local minima in the transmembrane state. This 1D profile was obtained with collective variable defined as the distance of the center of mass of the peptide to the center of mass of the membrane. (*A*) The free energy profile for L12 peptide and (*B*) for L16 peptide. The shaded region indicates the standard error of the mean.

DISCUSSION

This work investigated the influence of lipid unsaturation on the peptide-membrane interactions with a particular focus on membrane curvature.

First, we verified that membranes with an increasing degree of unsaturation exhibit increased membrane fluidity, larger average areas per lipid, decreased order of lipid tails, and more pronounced lipid-packing defects. These structural alterations of membrane enhance its fluidity and permeability, significantly impacting interactions with surrounding molecules and proteins (40).

Next, we examined how peptides respond to changes in membrane properties. Both mean hydrophobicity of the

TABLE 2 The free energy difference ($\Delta\Delta G$) in kJ/mol between the adsorbed and transmembrane state of the peptides in the POPC, DOPC, and PAPC membranes

Membrane	L12	L16
POPC	52.7 ± 1.3	19.8 ± 0.6
DOPC	47.0 ± 2.3	12.0 ± 0.1
PAPC	23.3 ± 0.1	4.2 ± 0.2

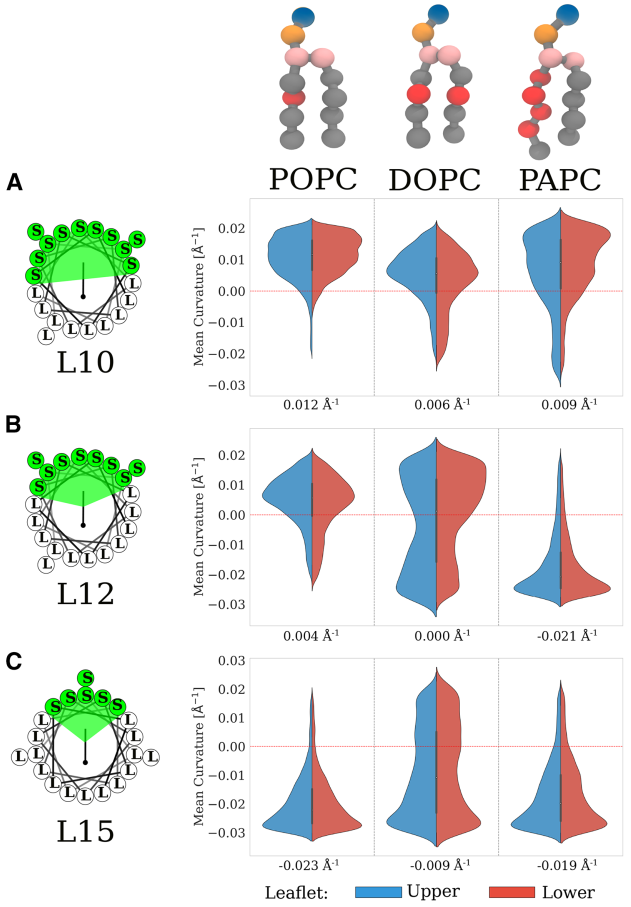

FIGURE 5 The curvature preference of the peptides L10, L12, and L15 on the POPC, DOPC, and PAPC membranes. The curvature preference of (*A*) L10, (*B*) L12, and (*C*) L15 peptides. The numbers associated with each violin plot represent the average mean curvature sensed by the peptide, which decreased with increasing membrane unsaturation and peptide hydrophobicity.

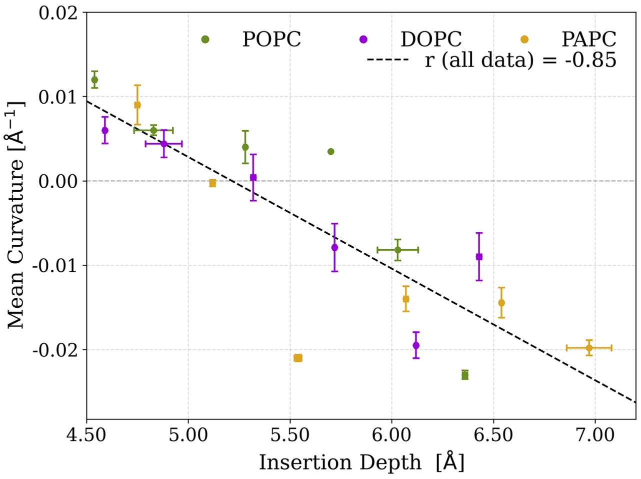

FIGURE 6 The correlation plot shows the relationship between the sensed mean curvature on buckled membranes by the tested peptides and their insertion depth on flat membranes, which in turn correlates with their insertion on buckled membranes (Fig. S14). The error bars represent the standard deviation computed from the mean curvatures of the upper and lower leaflets. Each point represents the mean of the curvature distribution for a specific peptide (L10–L15) across the three membrane compositions: POPC ($r = -0.90$), DOPC ($r = -0.87$), and PAPC ($r = -0.78$).

peptides and increased unsaturation of lipid membranes led to deeper insertion of the peptides. The deeper peptide insertion in unsaturated membranes appears to be influenced by the lower order parameters of the acyl chains (Fig. S4) and a reduction in the bilayer midplane pressure associated with unsaturation (42), as evidenced by the lateral pressure profiles presented in Fig. S13. However, note that the observed deeper peptide insertion for more unsaturated membranes could be specific to our simplified peptides (using only leucine and serine residues), and more complex peptides could have the opposite behavior (43).

For the model peptides employed, composed of leucine and serine residues only, we observed a linear relationship between their mean hydrophobicity and insertion depth. The strong correlation is likely a result of simplicity of the model peptides, and for chemically diverse peptides, the relationship may become nonlinear as previously reported (22).

Having established the relation between deeper peptide insertion and increasing unsaturation, we investigated how the membrane-embedded helices of transmembrane proteins may be affected. Very hydrophobic α-helices, e.g., the L16 peptide, adopted a dynamic topological orientation, challenging the conventional view that membrane protein topology is static. In the biological context, proteins in the secretory pathway can respond differently to membrane curvature and lipid composition. It is, therefore, plausible that the same peptide could sense curvature when bound peripherally to the plasma membrane but adopt a transmembrane orientation in the ER (44).

We focused on single α-helical peptides and the energetics of their reorientation in membranes with different unsaturation. The results indicate that the peptide's hydrophobic properties and the lipid environment significantly influence the energetic barrier for peptide reorientation. Given that the acyl chains of lipids are inherently hydrophobic, the L16 peptide, which exhibits the greatest hydrophobicity out of the peptides tested here, was more readily able to adopt a transmembrane orientation compared with the less hydrophobic L12 peptide. Extending these findings, we observed that some peptides adopted a metastable transmembrane state in unsaturated membranes. The enhanced tendency for peptide reorientation observed in polyunsaturated membranes may result from the presence of double bonds in unsaturated lipids. These double bonds introduce local polarity differences, increased fluidity, and reduced midplane pressure, thus promoting energetically favorable peptide interactions. Additionally, the energy barrier for peptide reorientation from a surface-adsorbed to a transmembrane state was lower for more hydrophobic peptides, since the membrane core provides a more hydrophobic environment

compared with the interface. These findings are consistent with the observations of Kabelka and Vácha, who demonstrated that peptides of varying lengths and hydrophobicities translocate through membranes via similar pathways but encounter different free-energy barriers depending on their specific properties (45).

These variations in topology can lead to the protein having different roles and functions. Indeed, the work by Vitrac et al. on changing lipid environment for LacY permease protein demonstrated how depletion of phosphatidylethanolamine lipids in the membrane led to the flipping of α-helical transmembrane domain to the periplasm (13). A similar dynamic reorientation of the N-terminus of the EmrE protein, belonging to the small multidrug-resistant protein family, has been observed to occur from a peripheral to a transmembrane state, depending on its dimerization state and sequence (23).

We tested peripheral peptides with varying hydrophobicity that could sense the unsaturation of the lipids on curved bilayers. The prevailing literature primarily associates curvature sensing with lipid-packing defects and the detection of positive curvatures. However, a broader mechanism for curvature sensing can be linked to the detection of internal stresses within membranes, as described by Campelo and Kozlov (20). In regions of negatively curved membrane regions, the lipid tail-order decreases (46). This may facilitate the accommodation of deeply inserting peptides, and consequently, deeply inserting peptides might preferentially localize to areas of negative curvature. Indeed, it has been demonstrated recently that amphipathic peptides are capable of sensing both positive and negative membrane curvatures (22).

In the case of the L15 peptide, its higher hydrophobicity enabled a deeper insertion into the membrane compared with the L10 and L12 peptides, resulting in the sampling of negative curvatures across all membrane types (POPC, DOPC, and PAPC). In contrast, the L12 peptide, with a more balanced amphipathic nature, exhibited different curvature preferences that were dependent upon the membrane composition. Specifically, the L12 peptide localized to positive curvatures on POPC, whereas it localized to negative curvatures on PAPC and exhibited no clear curvature preference on DOPC membrane (Fig. 5). Consequently, we hypothesize that peptides with properties similar to the L12 peptide would be capable of sensing membrane unsaturation and partitioning themselves into membrane regions with different curvatures depending on lipid composition. In our case, the L12 peptide showed the most interesting sensing behavior. It changed its curvature preference across POPC, DOPC, and PAPC bilayers. Therefore, we could characterize peptides with properties similar to L12 as sensors of unsaturation. Our work hints that the protein localization could change as a response to the alteration of the surrounding lipid environment or as a consequence of a single-point mutation, altogether hindering the protein's biological function (9).

CONCLUSION

We investigated the intricate relationship between peptide hydrophobicity, membrane unsaturation, and curvature effects using Martini 2 coarse-grained simulations. The results show that highly hydrophobic peptides, such as the L16 peptide, insert deeper into lipid membranes and can reorient themselves in the metastable transmembrane state in unsaturated membranes. These observations emphasize the enhanced interactions facilitated by greater degree of unsaturation, which promotes both deeper insertion and transmembrane orientation.

Building on these insights into peptide insertion and orientation, our study further demonstrates how membrane unsaturation affects peptide preference for curved membranes. More hydrophilic peptides preferentially sense and localize to positive curvatures, whereas very hydrophobic peptides are inclined toward negative curvatures. The peptides with a particular level of amphipathicity, such as L12 peptide, displayed curvature sensing dependent on membrane unsaturation, preferring positive curvature on saturated membranes and negative curvature on unsaturated membranes. This suggests that peptide hydrophobicity not only influences insertion depth and orientation but also controls the ability to sense membrane curvature and unsaturation. However, further investigations are required to ascertain whether our findings persist in bilayer models that display the interleaflet asymmetry characteristic of the plasma membrane and in complex lipid mixtures with varying degrees of unsaturation.

Together, these findings provide valuable insights into peptide-membrane interactions and may inform the design of membrane-active peptides for therapeutic and biotechnological applications. Future research could extend these findings to more complex transmembrane proteins and their lipid composition dependent behavior.

DATA AND CODE AVAILABILITY

The simulation trajectories and input files are available on Zenodo (https://doi.org/10.5281/zenodo.17131781). Due to storage limitations, the trajectories were downsampled, and only every 10th frame was saved, compared with the original trajectories.

ACKNOWLEDGMENTS

This work was supported by the European Research Council (ERC) under the European Union's Horizon 2020 research and innovation programme (grant agreement no. 101001470), the project National Institute of Virology and Bacteriology (Program EXCELES, ID project no. LX22NPO5103), funded by the European Union – Next Generation EU, and the Research Council of Finland (postdoctoral researcher grant no. 338160 for M.J.). Computational resources were provided by the CESNET, CERIT Scientific

Cloud, IT4 Innovations National Supercomputing Center by MEYS CR through the e-INFRA CZ (ID: 90254), and CSC – IT Center for Science (Espoo, Finland).

AUTHOR CONTRIBUTIONS

S.P. carried out all simulations, analyzed the data, and wrote the article. P.P. analyzed the data, assisted with the technical solutions, and wrote the article. M.J. assisted with technical solutions and wrote the article. R.V. designed the research and wrote the article.

DECLARATION OF INTERESTS

The authors declare no competing interests.

SUPPORTING MATERIAL

Supporting material can be found online at https://doi.org/10.1016/j.bpj.2025.09.042.

REFERENCES

1. Ian, M. A…., 2012. Regulating the regulator: post-translational modification of RAS. *Nat. Rev. Mol. Cell Biol.* 13:39–51.
2. Marco, A. O. M., and M. Glogauer. 2010. Pivotal Advance: Phospholipids determine net membrane surface charge resulting in differential localization of active Rac1 and Rac2. *J. Leukoc. Biol.* 87:545–555.
3. Joost, H., and P. L. Tim. 2005. Lipid traffic: floppy drives and a superhighway. *Nat. Rev. Mol. Cell Biol.* 6:209–220.
4. Bigay, J., P. Gounon, …, B. Antonny. 2003. Lipid packing sensed by ArfGAP1 couples COPI coat disassembly to membrane bilayer curvature. *Nature.* 426:563–566.
5. Bigay, J., J. F. Casella, …, B. Antonny. 2005. ArfGAP1 responds to membrane curvature through the folding of a lipid packing sensor motif. *EMBO J.* 24:2244–2253.
6. Frost, A., V. M. Unger, and P. De Camilli. 2009. The BAR domain superfamily: membrane-molding macromolecules. *Cell.* 137:191–196.
7. Bruno, A…., 1997. Activation of ADP-ribosylation factor 1 GTPase-activating protein by phosphatidylcholine-derived diacylglycerols. *J. Biol. Chem.* 272:30848–30851.
8. Levi, S., M. Rawet, …, D. Cassel. 2008. Topology of amphipathic motifs mediating Golgi localization in ArfGAP1 and its splice isoforms. *J. Biol. Chem.* 283:8564–8572.
9. Lorent, J. H., K. R. Levental, …, I. Levental. 2020. Plasma membranes are asymmetric in lipid unsaturation, packing and protein shape. *Nat. Chem. Biol.* 16:644–652.
10. Schneiter, R., B. Brügger, …, S. D. Kohlwein. 1999. Electrospray ionization tandem mass spectrometry (ESI-MS/MS) analysis of the lipid molecular species composition of yeast subcellular membranes reveals acyl chain-based sorting/remodeling of distinct molecular species en route to the plasma membrane. *J. Cell Biol.* 146:741–754.
11. Tuller, G., T. Nemec, …, G. Daum. 1999. Lipid composition of subcellular membranes of an FY1679-derived haploid yeast wild-type strain grown on different carbon sources. *Yeast.* 15:1555–1564.
12. Mellman, I., and W. J. Nelson. 2008. Coordinated protein sorting, targeting and distribution in polarized cells. *Nat. Rev. Mol. Cell Biol.* 9:833–845.
13. Vitrac, H…., 2015. Dynamic membrane protein topological switching upon changes in phospholipid environment. *Proc. Natl. Acad. Sci.* 112.45:13874–13879.
14. Bigay, J., and B. Antonny. 2012. Curvature, lipid packing, and electrostatics of membrane organelles: defining cellular territories in determining specificity. *Dev. Cell.* 23:886–895.
15. Wade, F. Z…., 2018. Synergy between intrinsically disordered domains and structured proteins amplifies membrane curvature sensing. *Nat. Commun.* 9:4152.
16. Brian, J. P…., 2004. BAR domains as sensors of membrane curvature: the amphiphysin BAR structure. *Science.* 303.5657:495–499.
17. Cui, H., E. Lyman, and G. A. Voth. 2011. Mechanism of membrane curvature sensing by amphipathic helix containing proteins. *Biophys. J.* 100:1271–1279.
18. Vanni, S., H. Hirose, …, R. Gautier. 2014. A sub-nanometre view of how membrane curvature and composition modulate lipid packing and protein recruitment. *Nat. Commun.* 5:4916.
19. Vamparys, L., R. Gautier, …, P. F. J. Fuchs. 2013. Conical lipids in flat bilayers induce packing defects similar to that induced by positive curvature. *Biophys. J.* 104:585–593.
20. Campelo, F., and M. M. Kozlov. 2014. Sensing membrane stresses by protein insertions. *PLoS Comput. Biol.* 10:e1003556.
21. Ollila, O. H. S., H. J. Risselada, …, S. J. Marrink. 2009. 3D pressure field in lipid membranes and membrane-protein complexes Physical Review Letters. *Phys. Rev. Lett.* 102:078101.
22. Pajtinka, P., and R. Vácha. 2023. Amphipathic Helices Can Sense Both Positive and Negative Curvatures of Lipid Membranes. *J. Phys. Chem. Lett.* 15:175–179.
23. Seurig, M., M. Ek, …, N. Fluman. 2019. Dynamic membrane topology in an unassembled membrane protein. *Nat. Chem. Biol.* 15:945–948.
24. Webb, B., and A. Sali. 2016. Comparative protein structure modeling using MODELLER. *Curr. Protoc. Bioinformatics.* 54:5–6.
25. Djurre, H. D. J…., 2013. Improved parameters for the martini coarse-grained protein force field. *J. Chem. Theor. Comput.* 9:687–697.
26. Gautier, R…., 2008. HELIQUEST: a web server to screen sequences with specific α-helical properties. *Bioinformatics.* 24.18:2101–2102.
27. Brooks, B. R., C. L. Brooks, 3rd, …, M. Karplus. 2009. CHARMM: the biomolecular simulation program. *J. Comput. Chem.* 30:1545–1614.
28. Jo, S., T. Kim, …, W. Im. 2008. CHARMM-GUI: a web-based graphical user interface for CHARMM. *J. Comput. Chem.* 29:1859–1865.
29. Abraham, M. J., T. Murtola, …, E. Lindahl. 2015. GROMACS: High performance molecular simulations through multi-level parallelism from laptops to supercomputers. *SoftwareX.* 1–2:19–25.
30. Bonomi, M., D. Branduardi, …, M. Parrinello. 2009. PLUMED: A portable plugin for free-energy calculations with molecular dynamics. *Comput. Phys. Commun.* 180:1961–1972.
31. Monticelli, L., S. K. Kandasamy, …, S. J. Marrink. 2008. The MARTINI coarse-grained force field: extension to proteins. *J. Chem. Theor. Comput.* 4:819–834.
32. Gómez-Llobregat, J., F. Elías-Wolff, and M. Lindén. 2016. Anisotropic membrane curvature sensing by amphipathic peptides. *Biophys. J.* 110:197–204.
33. Lindahl, V., J. Lidmar, and B. Hess. 2014. Accelerated weight histogram method for exploring free energy landscapes. *J. Chem. Phys.* 141:044110.
34. Bhaskara, R. M., P. Grumati, …, G. Hummer. 2019. Curvature induction and membrane remodeling by FAM134B reticulon homology domain assist selective ER-phagy. *Nat. Commun.* 10:2370.
35. Deserno, M. 2015. Fluid lipid membranes: From differential geometry to curvature stresses. *Chem. Phys. Lipids.* 185:11–45.
36. Kučerka, N., M.-P. Nieh, and J. Katsaras. 2011. Fluid phase lipid areas and bilayer thicknesses of commonly used phosphatidylcholines as a function of temperature. *Biochim. Biophys. Acta Biomembr.* 1808.11:2761–2771.
37. Kučerka, N…., 2008. Lipid bilayer structure determined by the simultaneous analysis of neutron and X-ray scattering data. *Biophys. J.* 95:2356–2367.
38. Kannan, R…., 2005. Packing and viscoelasticity of polyunsaturated ω-3 and ω-6 lipid bilayers as seen by 2H NMR and X-ray diffraction. *J. Am. Chem. Soc.* 127:1576–1588.

39. Holte, L. L., S. A. Peter, …, K. Gawrisch. 1995. 2H nuclear magnetic resonance order parameter profiles suggest a change of molecular shape for phosphatidylcholines containing a polyunsaturated acyl chain. *Biophys. J.* 68:2396–2403.

40. Harayama, T., and H. Riezman. 2018. Understanding the diversity of membrane lipid composition. *Nat. Rev. Mol. Cell Biol.* 19:281–296.

41. Boyd, K. J., N. N. Alder, and R. M. Eric. 2017. Buckling under pressure: curvature-based lipid segregation and stability modulation in cardiolipin-containing bilayers. *Langmuir.* 33:6937–6946.

42. Ollila, S., M. T. Hyvönen, and I. Vattulainen. 2007. Polyunsaturation in lipid membranes: dynamic properties and lateral pressure profiles. *J. Phys. Chem. B.* 111:3139–3150.

43. Perrin, B. S., A. J. Sodt, …, R. W. Pastor. 2015. The curvature induction of surface-bound antimicrobial peptides piscidin 1 and piscidin 3 varies with lipid chain length. *Biophys. J.* 108:498a.

44. Bogdanov, M., W. Dowhan, and H. Vitrac. 2014. Lipids and topological rules governing membrane protein assembly. *Biochim. Biophys. Acta.* 1843:1475–1488.

45. Kabelka, I., and R. Vácha. 2018. Optimal hydrophobicity and reorientation of amphiphilic peptides translocating through membrane. *Biophys. J.* 115:1045–1054.

46. Risselada, H. J., and S. J. Marrink. 2009. Curvature effects on lipid packing and dynamics in liposomes revealed by coarse grained molecular dynamics simulations. *Phys. Chem. Chem. Phys.* 11:2056–2067.

Biophysical Journal, Volume *124*

Supplemental information

Helical sensors of membrane saturation: Changes in orientation and curvature preference

Sushmita Pal, Peter Pajtinka, Matti Javanainen, and Robert Vácha

Helical Sensors of Membrane Saturation: Changes in Orientation and Curvature Preference

Sushmita Pal[1,2], Peter Pajtinka[1,2], Matti Javanainen[4,5], and Robert Vácha[1,2,3,*]

[1]CEITEC – Central European Institute of Technology, Masaryk University, Kamenice 753/5, 625 00 Brno, Czech Republic
[2]National Centre for Biomolecular Research, Faculty of Science, Masaryk University, Kamenice 5, 625 00 Brno, Czech Republic
[3]Department of Condensed Matter Physics, Faculty of Science, Masaryk University, Kotlářská 267/2, 611 37 Brno, Czech Republic
[4]Unit of Physics, Tampere University, FI-33720 Tampere, Finland
[5]Institute of Biotechnology, University of Helsinki, FI-00790 Helsinki, Finland
[*]Correspondence: robert.vacha@muni.cz

SUPPLEMENTARY METHODS

Insertion Depth

The system with planar membrane comprised of 184 lipid molecules (92 per leaflet) and two peptide copies, one copy placed on each leaflet in the headgroup region with parallel orientation to the membrane plane. The system contained approximately 3,000 water beads. To mimic physiological conditions, Na and Cl ions were added at a 0.15 M concentration. The energy of the system was minimized by the steepest descent algorithm.

The system preparation proceeded with a 100 ns equilibration run with a timestep of 20 femtoseconds, where the temperature was kept at 310 K using a stochastic velocity rescaling thermostat (1) with a coupling constant of 1 ps. The pressure was maintained at 1 bar using the Berendsen (2) barostat with semi-isotropic coupling and a coupling constant of 5 ps using a compressibility of $3{\times}10^{-4}$ bar^{-1}. Electrostatic interactions were screened by a dielectric constant of 15, and treated using a reaction field method for speed (3), although long-range contributions were eliminated by setting $\epsilon_{RF} = \infty$ beyond a cutoff radius of 1.1 nm. The cutoff radius for the van der Waals interactions was also set to 1.1 nm. The molecular dynamics simulations were performed at temperature 310 K using the stochastic velocity rescaling algorithm (1) with a time constant of 1 ps. Semi-isotropic Parinello–Rahman (4) barostat was used to maintain the pressure of 1 bar with coupling constant of 12 ps and a compressibility set to $3{\times}10^{-4}$ bar^{-1}. The rest of the settings were identical to those used in the NPT equilibration.

The reported lipid packing defects (Figure S3) were computed using the PackMem package (5). The values represent the total defect area calculated from Martini coarse-grained simulations, reflecting two-dimensional surface defects where hydrophobic regions are transiently exposed to the solvent (5). While the CG defect areas computed using the Martini force field exhibit a qualitative correlation with defect trends observed in all-atom simulations, the absolute values of the defect areas are not directly comparable. This is due to the intrinsic resolution limitation of CG models, where each bead represents multiple atoms, leading to spatial averaging. Therefore, the defect area computed in Martini simulations should be interpreted as an indicative metric, useful for comparative analysis across systems, but not as a precise quantitative measure of solvent-exposed area.

We have analyzed spatially resolved properties—including bilayer thickness and lipid order parameters—around the peptide insertion site. The thickness was calculated with considering a local cylinder around the peptide of radius 2.5 nm and then the thickness was measured by taking an average z-distance between the phosphate beads between the two leaflets. The order parameter was calculated using the gorder package to see the local effects of peptide on altering the order parameter of the lipids (6).

Free energy calculations

A planar system with 400 lipids of type POPC, DOPC and PAPC were using CHARMM-GUI web server. These membranes were then subjected to energy minimization employing the steepest descent algorithm, followed by an equilibration protocol with varying timesteps and duration, (i) 2 fs and 1 ns, (ii) 5 fs and 1 ns, (iii) 10 fs and 0.1 ns, (iv) 15 fs and 0.45 ns, and (v) 20 fs and 0.1 ns and a longer equilibration of 3 ns with 20 fs timestep. Prior to the addition of peptides, both solvent and ions were removed from the system. One peptide was placed on upper membrane leaflet. Subsequently, the system was resolvated with approximately 12,000 water beads and ions were added to reach the concentration of 0.15 M NaCl, replicating physiological conditions.

The energy minimization of the system was performed again using the steepest descent algorithm, setting the total energy tolerance to 100 kJ mol^{-1} nm^{-1}. This was followed by a two-step equilibration process: initially, a short 1 ns equilibration during which the peptides were pulled toward the membrane surface using a harmonic potential with cylindrical geometry applied to the backbone of the peptide beads, to prevent sampling of peptide in solution. This was followed by an extended 100 ns equilibration following the same equilibration protocol as for the basic planar membrane simulation.

Buckled Bilayer

The system construction started with a flat bilayer comprising 1008 lipids symmetrically distributed across both leaflets. The system was subsequently solvated with approximately 24,000 water beads, and Na and Cl ions were added to attain 0.15 M NaCl concentration. Energy minimization was conducted using the steepest-descent algorithm with a maximal force tolerance of 100 kJ mol^{-1} nm^{-1}. Equilibration proceeded through five stages following the equilibration protocol described above for the planar membrane.

After the initial short equilibration steps, a longer equilibration was carried out with a timestep of 20 fs over a duration of 300 ns, following the same parameters as in the production dynamics of planar membrane.

The membrane was then buckled using PLUMED moving restraints. First, a compressing moving restraint was applied to the box size in the y-direction resulting in a prolonged membrane patch in x-direction. Next, moving restraints were applied in the x-direction while keeping the box size in the y-direction constant, resulting in membrane buckling along x-axis. The extent of bilayer compression was determined by the compression strain γ (Eq. S1), where $L_{x,0}$ is the box length of the planar bilayer in the dimension of compression and $L_{x,i}$ is the box length of the buckled bilayer. A constant value of $\gamma = 0.14$ was used for all membranes.

$$\gamma = \frac{L_{x,0} - L_{x,i}}{L_{x,0}} \tag{S1}$$

The final buckled membranes were then equilibrated for 200 ns, while the buckled shape was preserved by setting the compressibility in x and y directions to 0 bar^{-1}. Water and ions were removed from buckled membrane before adding peptides. One peptide was placed on each membrane leaflet. The system was then re-solvated with ~30,000 water beads, and ions corresponding to the final 0.15 M NaCl concentration were added. The system was energy minimized using the steepest descent algorithm with a maximum force tolerance of 100 kJ mol^{-1}. The snapshot of the prepared system is shown in **??** in the main text.

The equilibration was performed in two steps. First, a short equilibration was performed with a time step of 10 fs and a duration of 1 ns. During this step, backbone beads were restrained by applying a harmonic potential, and later, a 100 ns long equilibration was performed with a time step of 20 fs without any applied biases. In both equilibration steps, the system temperature was kept at 310 K using stochastic velocity-rescaling thermostat (1) and kept at a reference pressure of 1 bar using Berendsen barostat (2) with coupling applied only in z-direction with compressibility set to 3×10^{-4} bar^{-1}. The equilibration steps were followed by production runs of at least 25 µs, using the Parrinello–Rahman barostat (4) with a coupling constant of 12 ps. All other parameters remained consistent with those used during the extended equilibration phase. The initial 2 µs of the simulated trajectories were excluded from the analysis.

Curvature reweighting

Membrane bending is symmetric with respect to the bilayer center. However, as in this work we focus on membrane surface, where there is an imbalance in area of positive and negative curvatures as reported previously (7). We tried to correct for this bias by evaluating the distribution of accessible curvature on membrane surface (as defined by phosphate beads) and used this distribution to reweight the distributions of sampled curvature by peptides using their respective abundance on the membrane surface.

First, we constructed a reference distribution of curvatures present on the membrane surface for membrane-only systems. All the analyzed trajectory frames were aligned with respect to the membrane buckled shape. Second, we used a simple (1D) Fourier series to fit the average membrane buckle, specifically its projection to XZ plane (as opposed to a 2D Fourier series used for frame-by-frame analysis of peptide curvature sensing). We did this to limit the effect of membrane fluctuations, which might make the analysis noisy. When considering the whole trajectory and average membrane shape, using a 1D Fourier series is reasonable as the curvature in the Y-direction (normal to the buckling direction) arises only due to membrane thermal undulations and averages out over time. We then obtained the total profile arc length of the membrane buckle and found 100 equidistant points along the arc length. These points were used to construct a mesh with equidistant Y-points, and at each node, mean curvature was calculated. This analysis was performed for every 100th trajectory frame, enabling us to construct a

distribution of accessible curvature at the membrane surface. A schematic of the procedure used is depicted in Figure S1.

To correct for the non-uniform sampling of curvature imposed by the membrane's buckled geometry, we first computed a reference curvature histogram from every 100th frame of the membrane-only system (for each lipid composition separately). This reference and each peptide's raw mean-curvature values were binned over the same interval of curvatures. For each bin i, we calculated a preliminary weight $w_i = \frac{p_i}{r_i}$, where p_i and r_i are the peptide and reference counts in that bin; bins representing $< 0.1\%$ of either distribution were set to zero to avoid artificial noise. The resulting weighted counts were then normalized, and inverse-transform sampling was used to draw new, bias-corrected curvature values in proportion to w_i. This procedure ensures that all peptides are compared while eliminating artifacts due to uneven sampling resulting from membrane curvature.

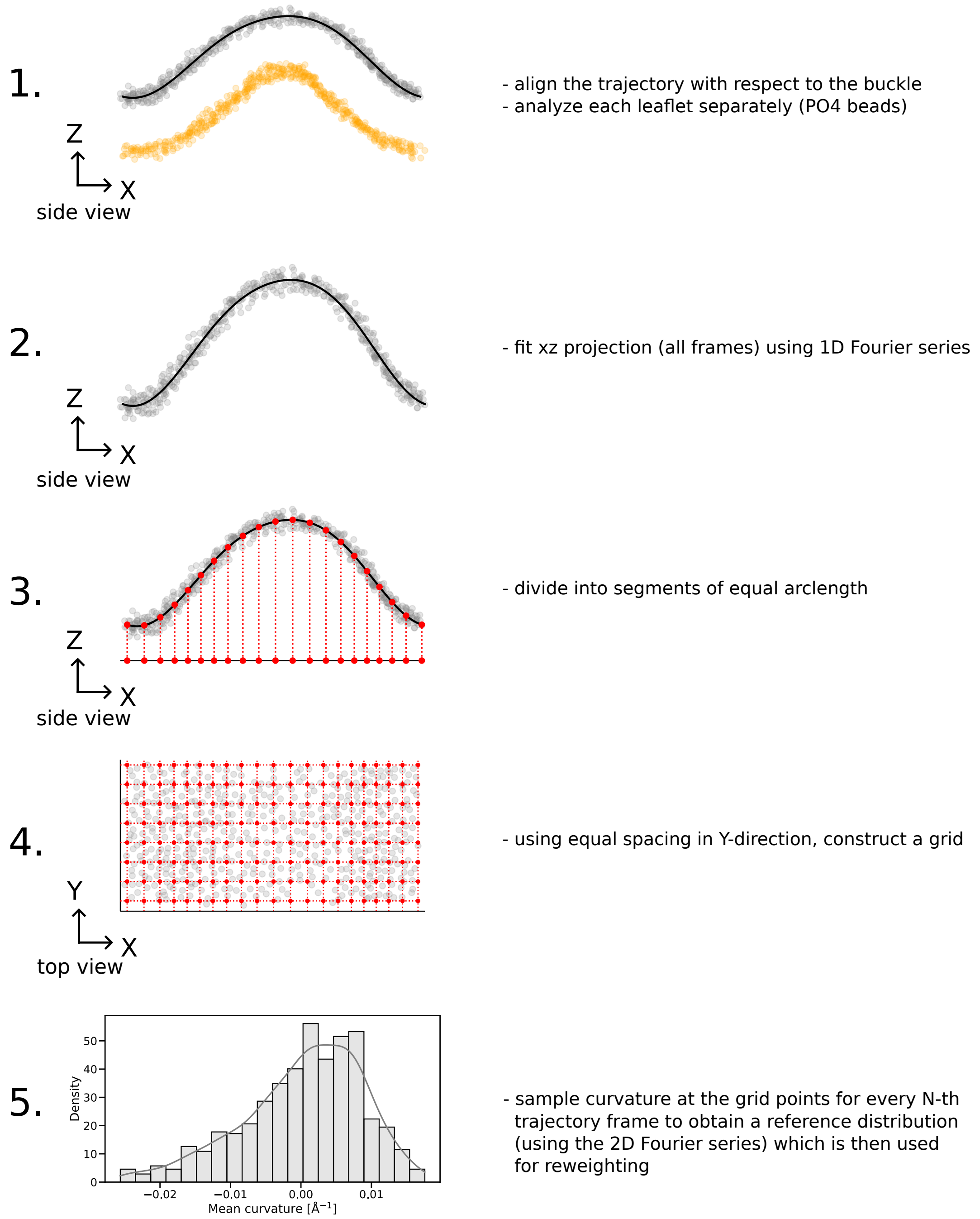

Figure S1: Schematic of the procedure used to obtain reference distributions for the curvature reweighting.

Lateral pressure profiles

To obtain lateral pressure profiles, we extended simulations of planar DOPC, POPC and PAPC membranes, as well as systems containing DOPC membrane and L10 or L15 peptides (by 5 µs. Positions and velocities of all atoms were saved every 500 ps. These trajectories were then post-processed using Gromacs-LS (8), to calculate lateral pressure profiles, $\pi(z)$, along the z-axis using Goetz-Lipowsky force decomposition (9) and 1 Å spacing.

To assess the curvature generating ability of the peptides, we calculated spontaneous monolayer curvature (C_0^m) of pure membrane and of membrane with peptides from planar membrane systems we used relation between the first moment of the lateral pressure profile and bending moment, $\kappa_m C_0^m$ (Eq. S2). In order to calculate C_0^m, knowledge of monolayer bending modulus, κ_m is necessary. Here, we used value of $5.9 \pm 0.2 \times 10^{-20}$J (from (10)).

$$\kappa_m C_0^m = \int_0^\infty z\,\pi(z)\,\mathrm{d}z \tag{S2}$$

Table S1: Spontaneous monolayer curvature (C_0^m) for DOPC membrane and DOPC membrane with peptides L10 and L15. For calculation of spontaneous curvature, monolayer bending modulus for DOPC of $5.9 \pm 0.2 \times 10^{-20}$J was used, based on (10). The error was determined from asymmetry of the lateral pressure profile.

Peptide	C_0^m [nm^{-1}]
DOPC	-0.168 ± 0.006
+L10	-0.167 ± 0.006
+L15	-0.178 ± 0.006

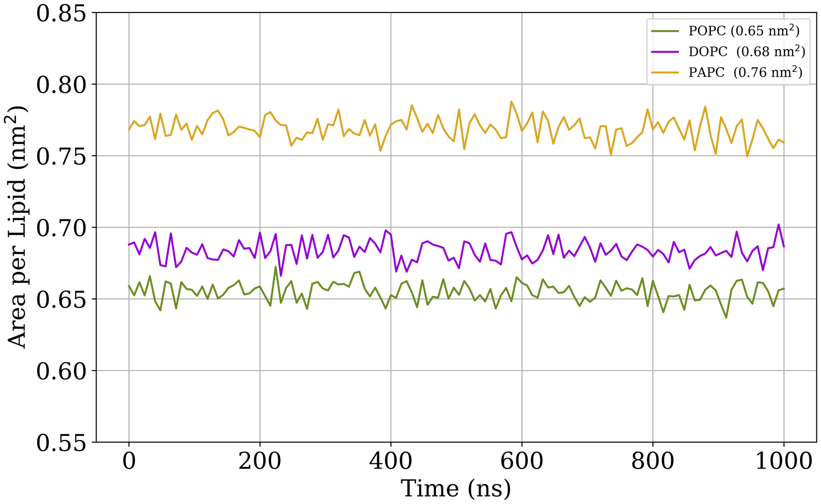

Figure S2: Area per lipid of POPC, DOPC, and PAPC planar membranes without peptides; the mean values are shown in the legend.

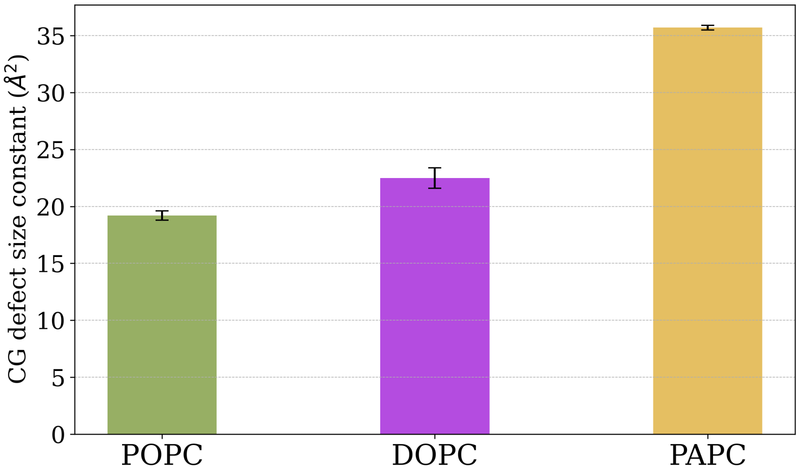

Figure S3: Area of lipid packing defects for the membranes of POPC, DOPC, and PAPC planar membranes using Martini model.

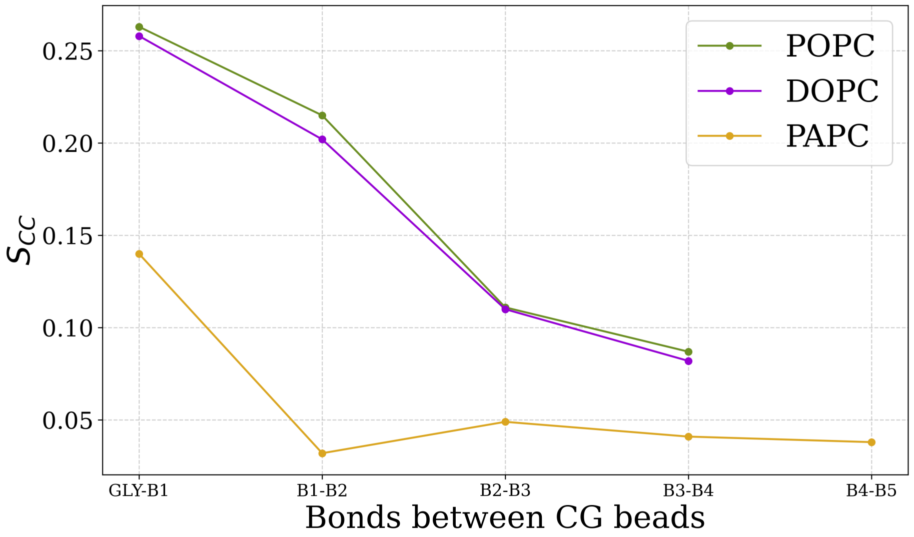

(a) S_{CC} Order parameters for sn-1 chain of POPC, DOPC and PAPC planar membranes.

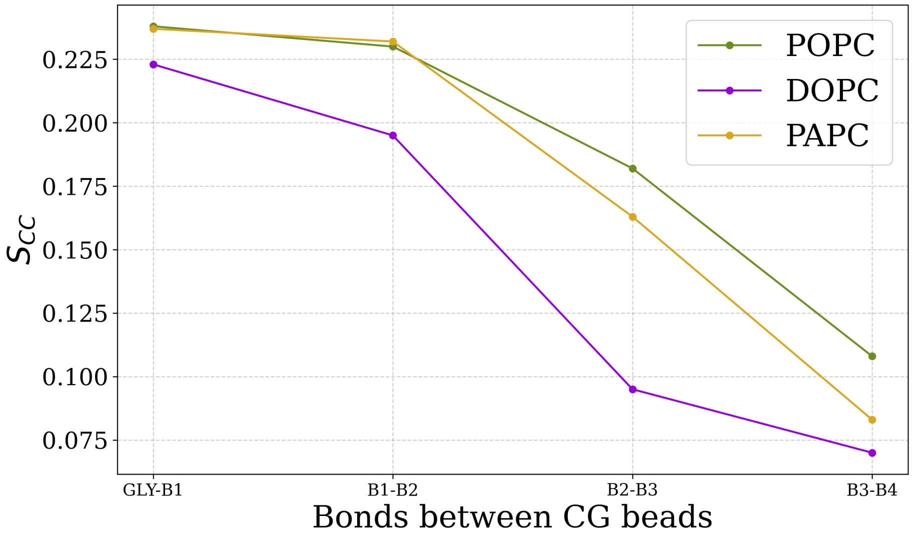

(b) S_{CC} Order parameters for sn-2 chain of POPC, DOPC and PAPC planar membranes.

Figure S4: S_{CC} Order parameters for sn-1 and sn-2 acyl chains of the POPC, DOPC, and PAPC planar membranes. GLY in the x-axis represent the glycerol bead and B1, B2, B3, B4 and B5 represent the beads along the acyl chains. a) Order paremeters of sn-1 acyl chain. B4-B5 beads is only valid for PAPC membrane b) Order Parameters of sn-2 acyl chain.

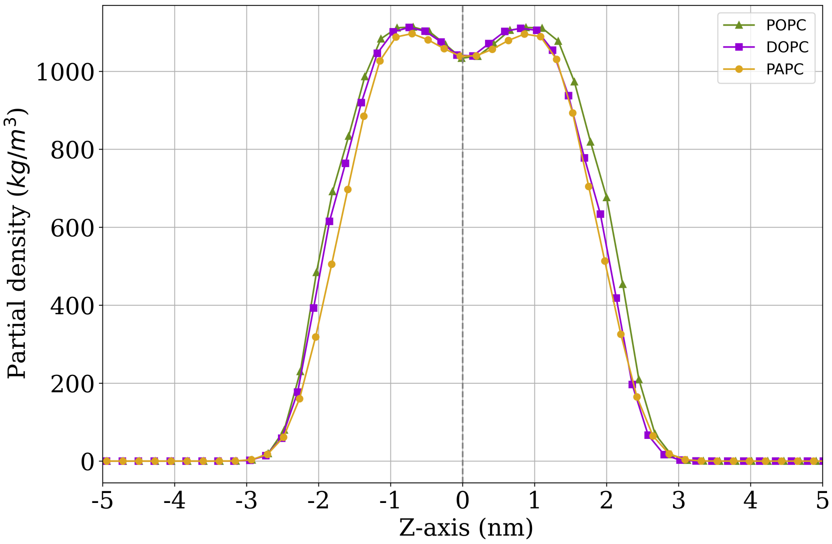

Figure S5: Partial density profiles of POPC, DOPC, and PAPC planar membranes.

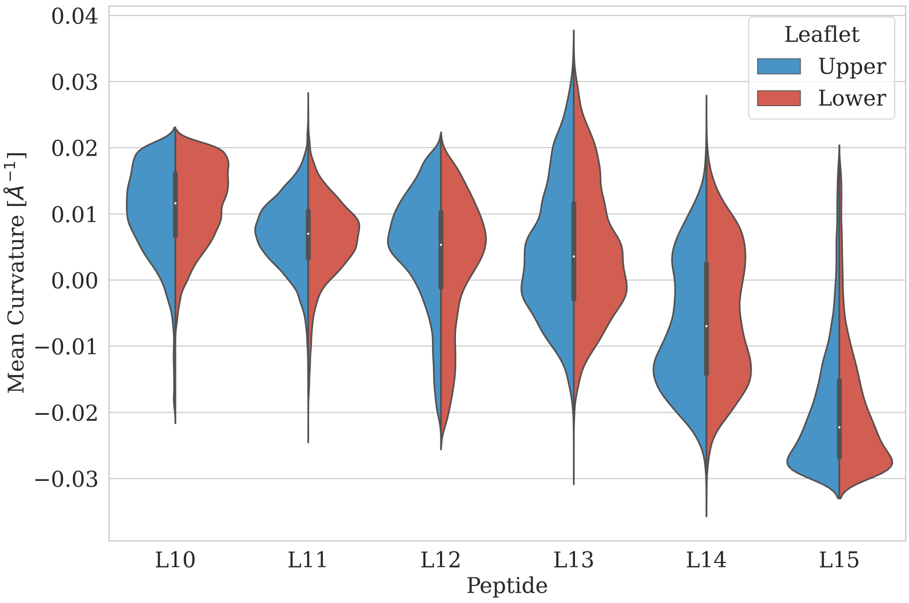

Figure S6: Reweighted mean curvature distribution for L10-L15 peptide on POPC buckled membrane.

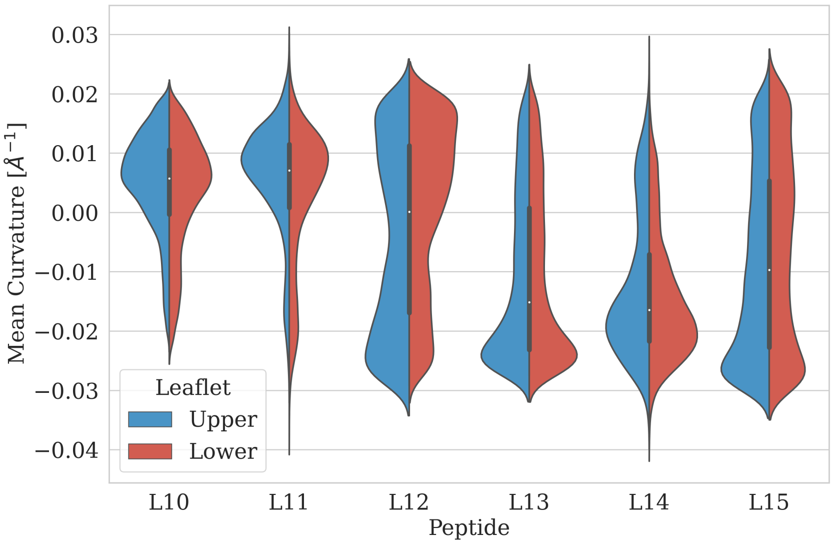

Figure S7: Reweighted mean curvature distribution for L10-L15 peptide on DOPC buckled membrane.

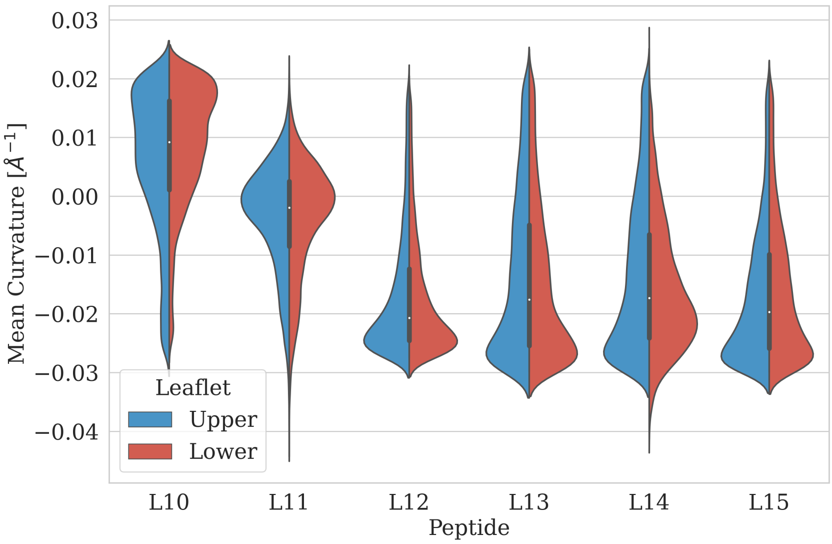

Figure S8: Reweighted mean curvature distribution for L10-L15 peptide on PAPC buckled membrane.

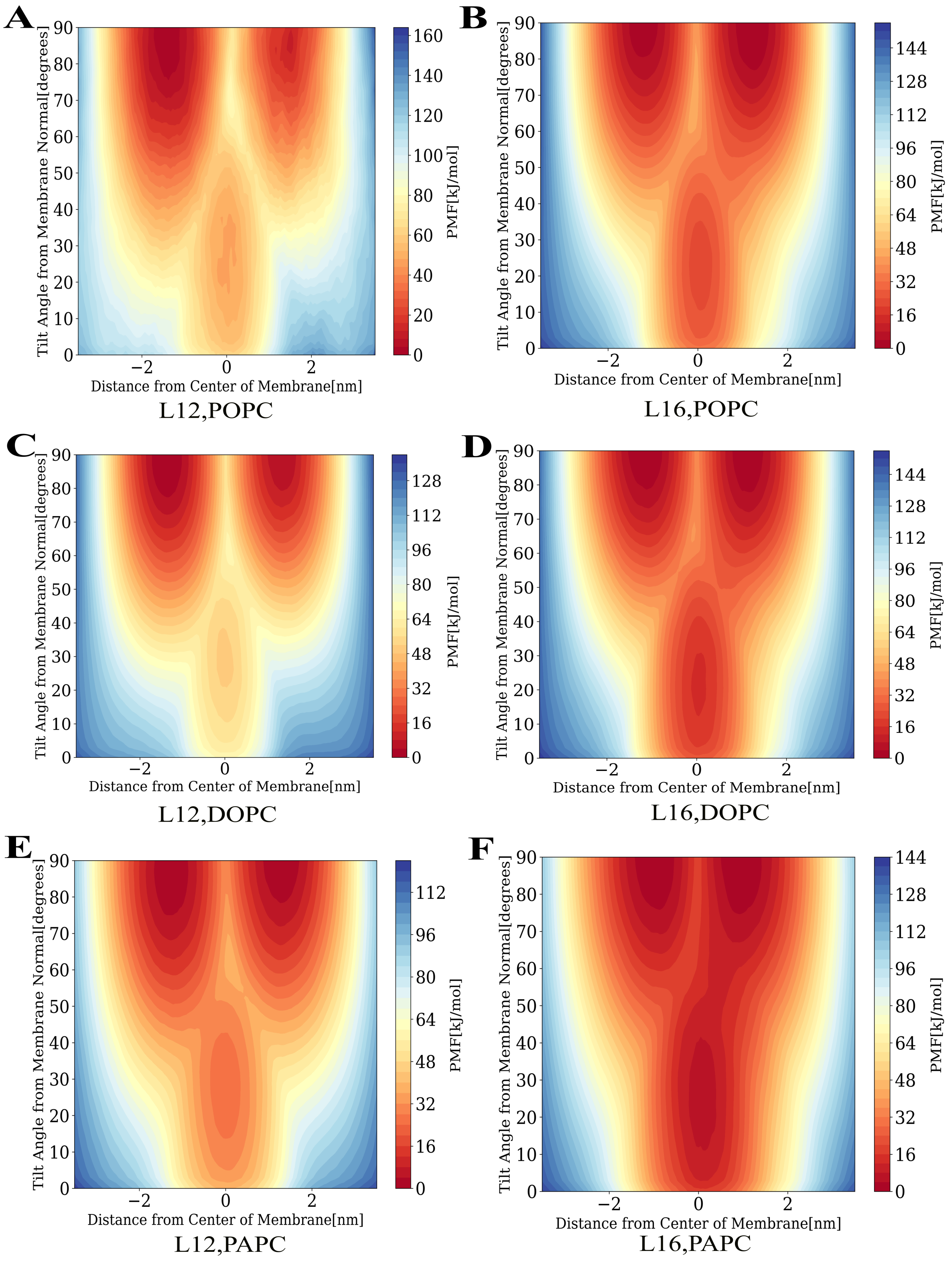

Figure S9: 2D Free energy profiles for the L12 and L16 peptides on POPC, DOPC, and PAPC planar membranes.

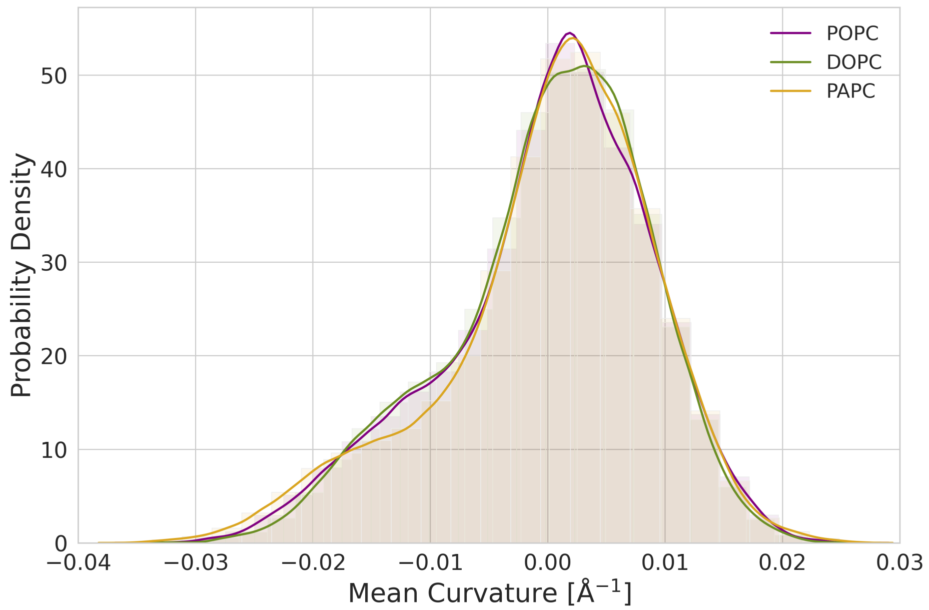

Figure S10: Accessible curvatures on POPC, DOPC, and PAPC buckled membranes.

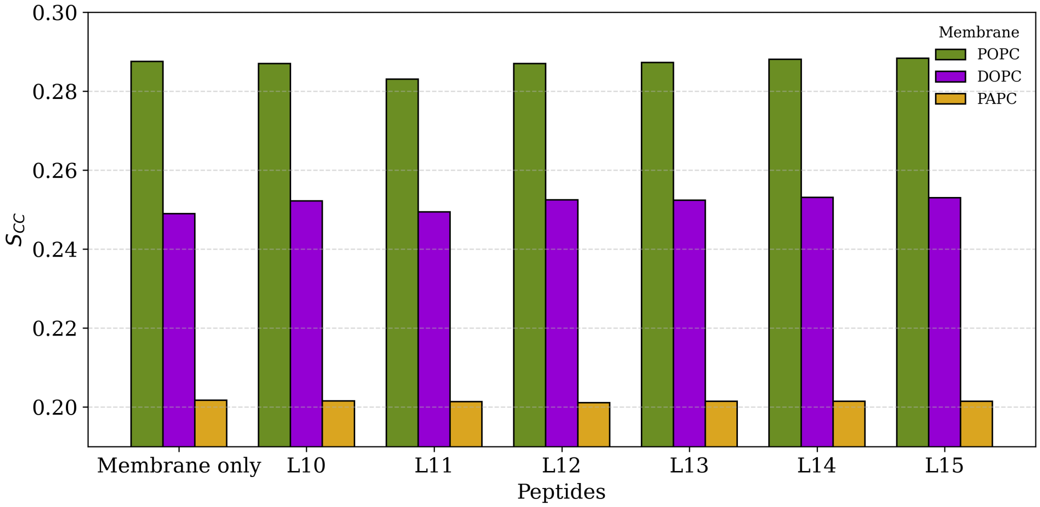

Figure S11: Average order parameters for POPC, DOPC and PAPC membrane within 2.5 nm of radius around the the peptides in the flat-membrane simulations.

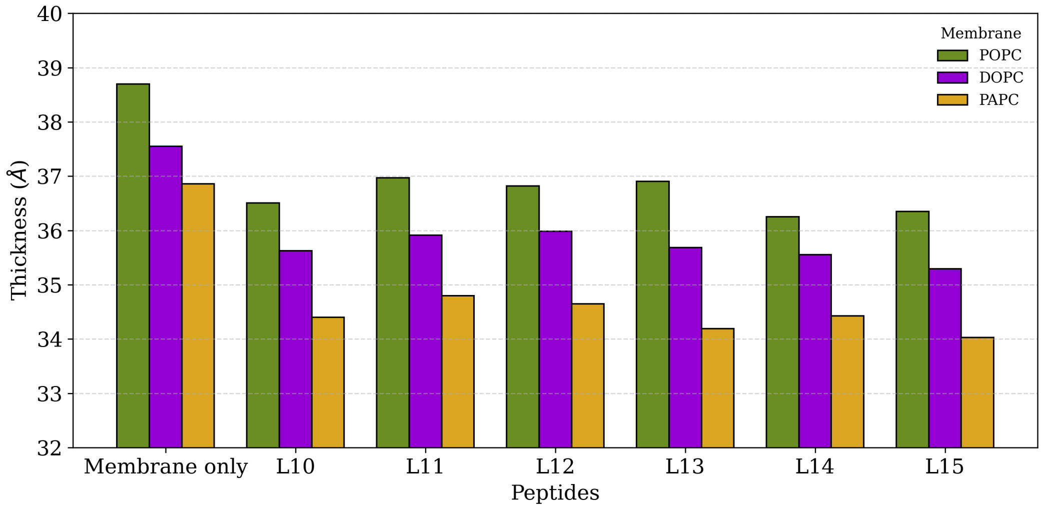

Figure S12: Average membrane thickness for POPC, DOPC and PAPC membrane within 2.5 nm around the peptide in the flat-membrane simulations.

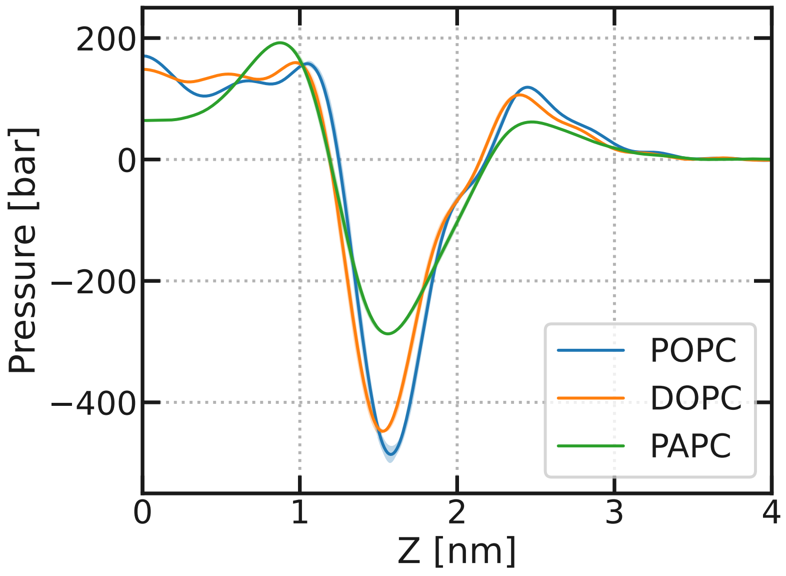

Figure S13: Lateral pressure profiles of POPC, DOPC, and PAPC membranes. As the profiles were symmetrized, only one leaflet is shown.

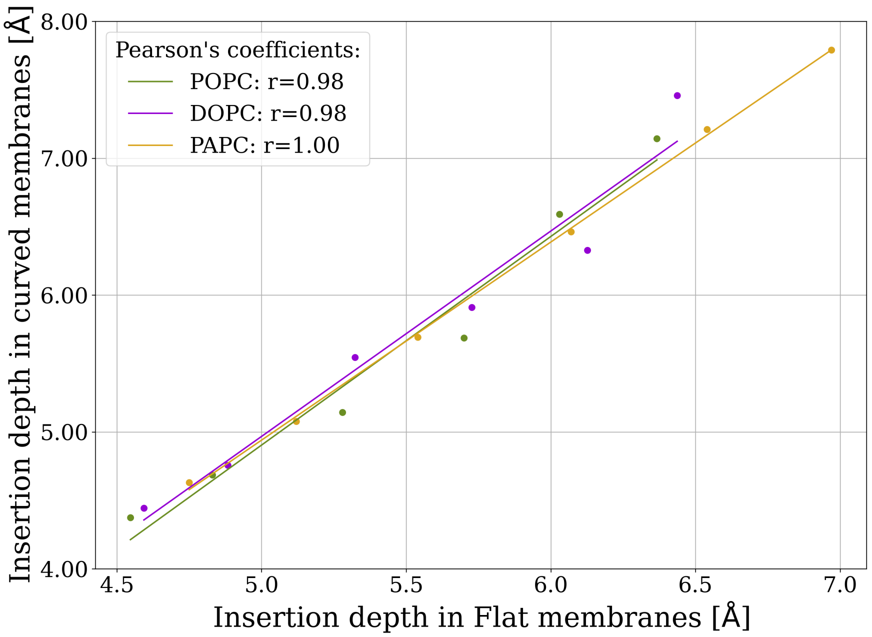

Figure S14: Correlation plot of peptide insertion depth in curved membrane against its insertion depth in planar bilayers.

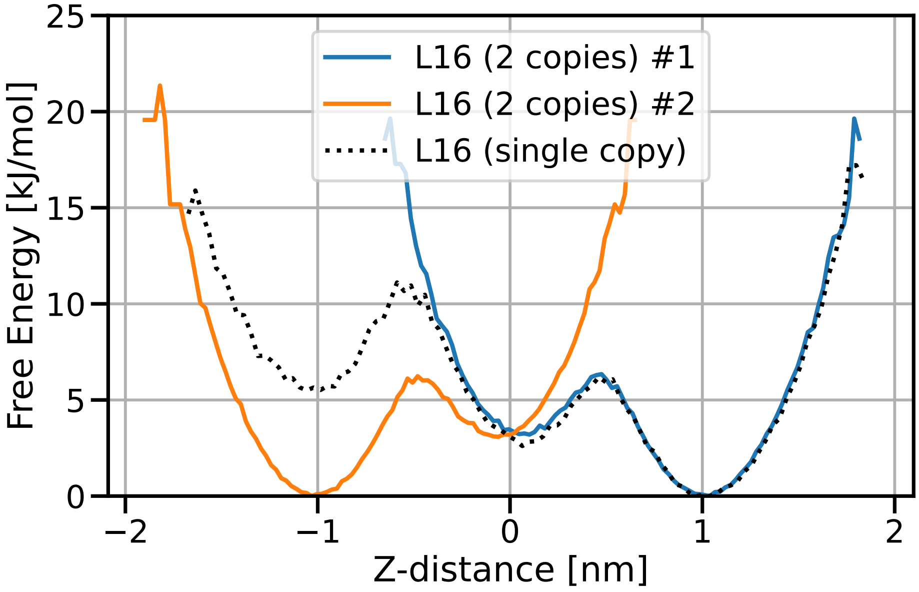

(a) The free energy profiles of peptide reorientation obtained from unbiased simulations on PAPC membrane with either single L16 peptide or one peptide copy on each leaflet (2 copies in total).

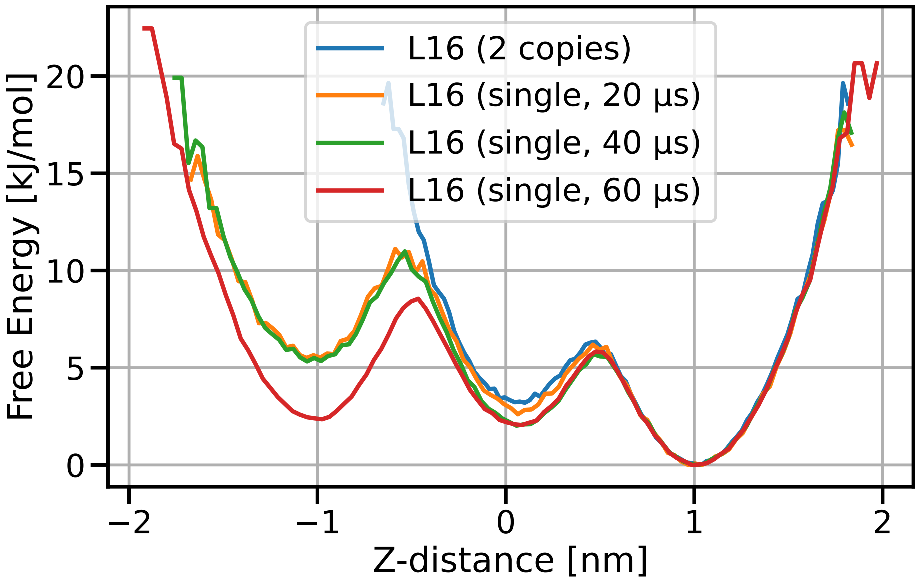

(b) The free energy profiles obtained from prolonged simulation of the system with a single L16 copy demonstrate the asymmetry of the unbiased simulation profile originated from the insufficient sampling within the used time scale.

Figure S15: Comparison of free energy profiles obtained from unbiased simulations containing two or a single copy of the L16 peptide.

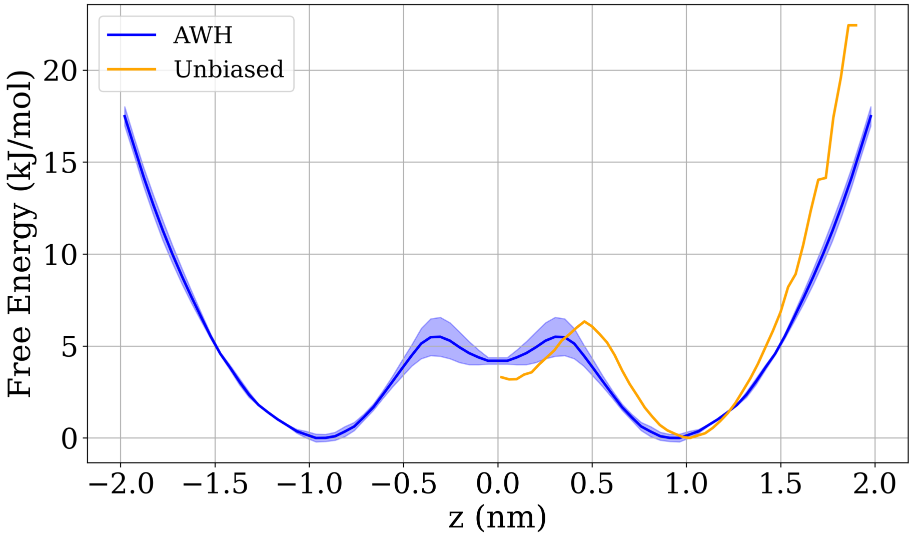

Figure S16: The plot shows the comparision of the free energy from unbiased simulations and AWH simulations for L16 peptide on PAPC membrane.

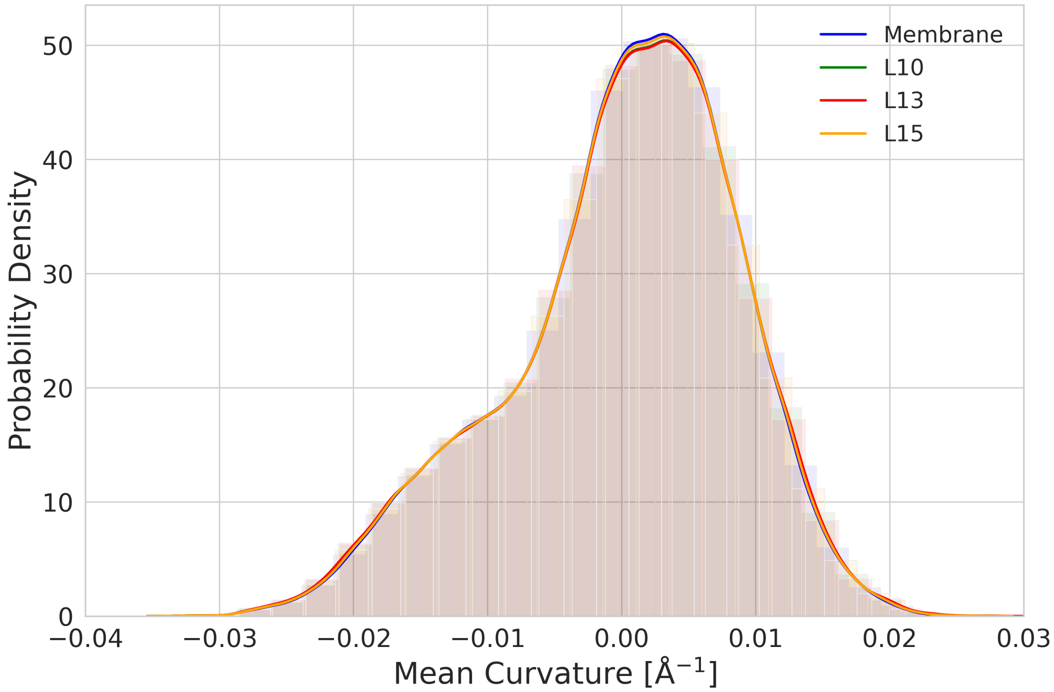

Figure S17: The accessible curvatures for L10, L12 and L15 peptide on DOPC membrane.

REFERENCES

1. Giovanni Bussi, Davide Donadio, and Michele Parrinello. "Canonical sampling through velocity rescaling". In: *The Journal of chemical physics* 126.1 (2007).
2. Herman JC Berendsen et al. "Molecular dynamics with coupling to an external bath". In: *The Journal of chemical physics* 81.8 (1984), pp. 3684–3690.
3. Djurre H De Jong et al. "Martini straight: Boosting performance using a shorter cutoff and GPUs". In: *Computer Physics Communications* 199 (2016), pp. 1–7.
4. Michele Parrinello and Aneesur Rahman. "Polymorphic transitions in single crystals: A new molecular dynamics method". In: *Journal of Applied physics* 52.12 (1981), pp. 7182–7190.
5. Romain Gautier et al. "PackMem: a versatile tool to compute and visualize interfacial packing defects in lipid bilayers". In: *Biophysical journal* 115.3 (2018), pp. 436–444.
6. Ladislav Bartoš, Peter Pajtinka, and Robert Vácha. "gorder: Comprehensive tool for calculating lipid order parameters from molecular simulations". In: *SoftwareX* 31 (2025), p. 102254. ISSN: 2352-7110. DOI: https://doi.org/10.1016/j.softx.2025.102254.
7. Kevin J Boyd, Nathan N Alder, and Eric R May. "Buckling under pressure: curvature-based lipid segregation and stability modulation in cardiolipin-containing bilayers". In: *Langmuir* 33.27 (2017), pp. 6937–6946.
8. Juan M Vanegas, Alejandro Torres-Sánchez, and Marino Arroyo. "Importance of force decomposition for local stress calculations in biomembrane molecular simulations". In: *Journal of chemical theory and computation* 10.2 (2014), pp. 691–702.
9. Rüdiger Goetz and Reinhard Lipowsky. "Computer simulations of bilayer membranes: self-assembly and interfacial tension". In: *The Journal of chemical physics* 108.17 (1998), pp. 7397–7409.
10. Richard M Venable, Frank LH Brown, and Richard W Pastor. "Mechanical properties of lipid bilayers from molecular dynamics simulation". In: *Chemistry and physics of lipids* 192 (2015), pp. 60–74.
